# Oral colonization of probiotics: one size fits all?

**DOI:** 10.1016/j.crmicr.2026.100642

**Published:** 2026-07-07

**Authors:** Wannes Van Holm, Julie Marynissen, Lien Van Campenhout, Yorick Minnebo, Fabian Mermans, Katalina Lauwens, Kobe Teughels, Mehraveh Saghi, Naiera Zayed, Nico Boon, Wim Teughels

**Affiliations:** a KU Leuven, Department of Oral Health Sciences, Periodontology and Oral Microbiology, Leuven, Belgium; b Ghent University (UGent), Centre for Microbial Ecology and Technology (CMET), Gent, Belgium; c Faculty of Pharmacy, Menoufia University, Shebeen El-Kom, Egypt; d University Hospitals Leuven, Dentistry service, Leuven, Belgium; e Chulalongkorn University, Faculty of Dentistry, Bangkok, Thailand; f Universiti Malaya, Department of Restorative Dentistry, Faculty of Dentistry, Kuala Lumpur, Malaysia

**Keywords:** Probiotics, Stomatognathic diseases, *Limosilactobacillus reuteri*, Bacterial adhesion, Precision medicine

## Abstract

The clinical application of probiotics for oral health is increasing, yet their colonization dynamics remain poorly understood. This study investigated whether administration timing, strain origin, and host-specific factors influence oral persistence.

Two *Limosilactobacillus reuteri* strains (oral vs. non-oral isolate) were evaluated in two in vivo studies. In the first, participants consumed probiotics either during the day or before bedtime; in the second, daily administration continued for 28 days, followed by a 7-day washout. Probiotic abundance was qPCR quantified and oral microbiomes were sequenced. Additionally, a retrospective analysis of periodontitis patients receiving probiotics was performed.

Bedtime administration significantly prolonged probiotic detectability compared to daytime intake. Both strains were largely transient, disappearing within days post-administration, though the oral isolate showed enhanced adhesion. Notably, a subset of participants retained the probiotic for up to a week, suggesting interindividual variability and potential biomarkers of colonization. Periodontitis patients who had higher concentrations of probiotics also displayed more improvement in pocket probing depth of deep pockets.

These findings highlight the need to consider timing, strain selection, and host factors in probiotic-based oral health interventions.

## Highlights

• Bedtime probiotic intake retains 100,000x more bacteria than daytime intake.
• Oral-origin *L. reuteri* strain colonizes better than the non-oral isolate.
• Probiotic persistence varies widely interindividually, some individuals retain strains 7+ days.
• Supragingival plaque shows highest proportional probiotic abundance by site.
• Higher probiotic levels correlate with greater pocket depth reduction in periodontitis.

## Graphical abstract

**Figure:**
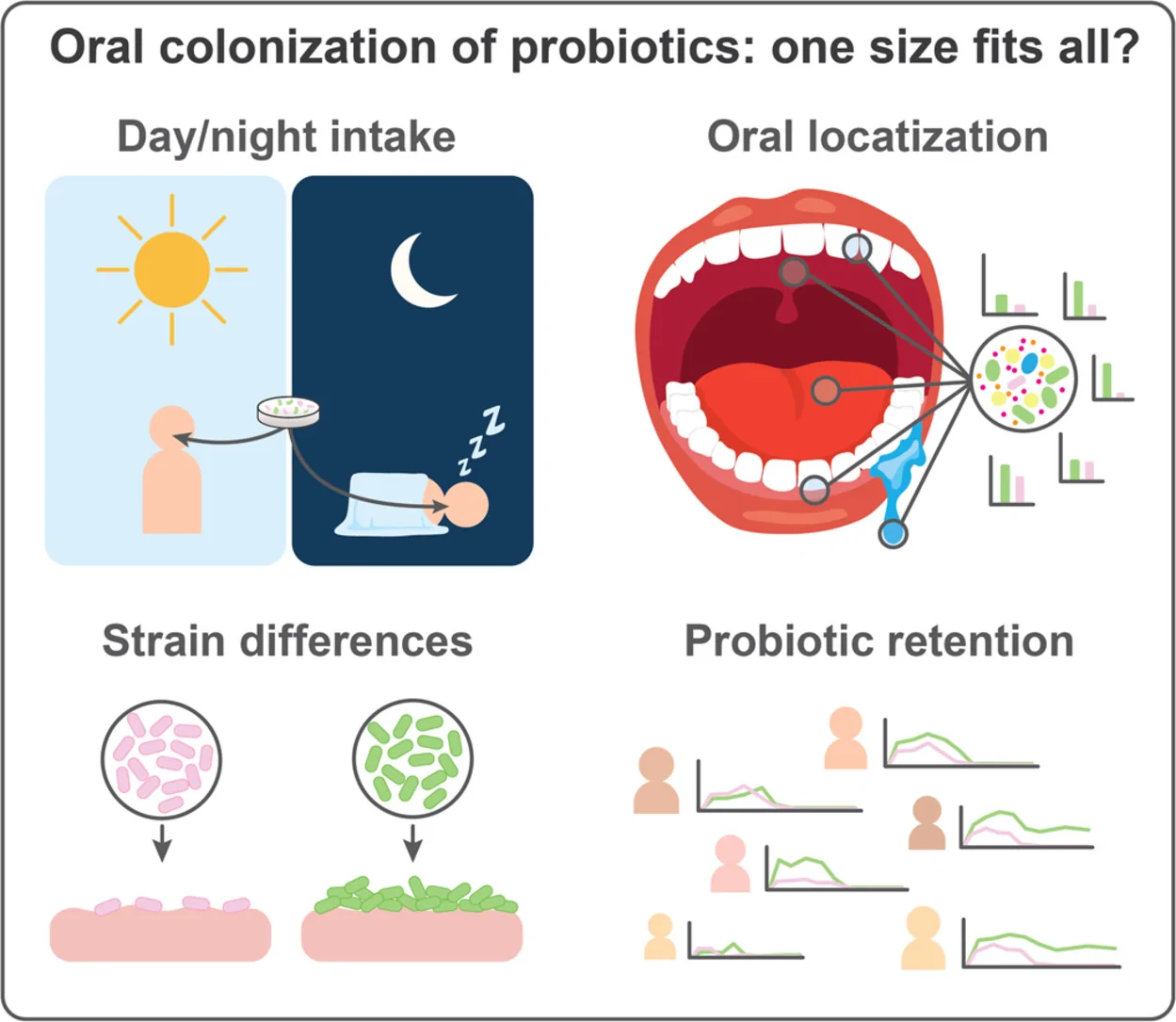

## Introduction

Probiotics are live microorganisms which, when administered in adequate amounts, confer a health benefit on the host (Hill et al., 2014). Over the past decade, their use for oral health has grown, and they have shown benefits for several oral diseases (Kiepś and Dembczyński, 2022; Twetman, 2018; Wilcox et al., 2019; Mundula et al., 2019).

The most common probiotic delivery method is lyophilised powders compressed into orally dissolvable lozenges (Van Holm et al., 2023). While from a business perspective, these are easy to produce and distribute, it might not be the optimal delivery method due to two interlinked limiting factors unique to probiotics for the oral cavity: salivary washout and the inactive nature of lyophilized bacteria. Contrary to gastrointestinal probiotics, which have more time to exert their probiotic mechanisms due to their slow transit through the GI tract, oral probiotics tend to only be minutes in the oral cavity due to the frequent swallowing of their carrier liquid, saliva.

Intertwined with the first factor is the inactive nature of the bacteria. While techniques such as lyophilization allow for longer shelf life (Kiepś and Dembczyński, 2022), the required inactivation of the probiotic also reduces their adhesive capabilities compared to fresh or reactivated probiotics (Van Holm et al., 2023). Aside from a perhaps inefficient delivery method, the oral microbiome is exceptionally resilient to outsiders (He et al., 2014; Zhou et al., 2013), further limiting the chances of a probiotic colonizing the oral cavity.

Despite probiotics for oral health being a growing field, with several articles being published every month, little is known on the colonization and retention of probiotics. The studies that do investigate the adhesion and retention of oral probiotics, the setups, tested probiotic strains and outcomes differ (Alforaidi et al., 2020; Chugh et al., 2020; Haukioja et al., 2006; Horz et al., 2007; Ibarlucea-Jerez et al., 2024; Yli-Knuuttila et al., 2006; Ravn et al., 2012; Sali et al., 2025). A better understanding of the dynamics of probiotic colonization, and where probiotics colonize the oral cavity can provide valuable insights on how to improve probiotic therapy for oral health.

The goal of the current study was to gain deeper insight in the colonization of the oral cavity of two commonly used probiotic *Limosilactobacillus reuteri* strains delivered through lozenges and how long they are retained after probiotic administration is ceased.

## Materials and methods

### Probiotic information & primer design

Prodentis® probiotic lozenges (BioGaia®, Lund, Sweden) containing both *Limosilactobacillus reuteri* ATCC PTA 5289 (LATCC) and *L. reuteri* DSM 17938 (LDSM) were used in this study.

Based on the available primers for LATCC and LDSM (Romani Vestman et al., 2013), and DNA sequences of the strains provided by BioGaia, dual labelled TaqMan® probes were designed to enhance sensitivity for the probiotics specifically (Table 1). The general probe rational was followed: 18–30 bp, probe close to either the forward or reverse primer, melting temperature 5–10 °C higher than primers, and no secondary structures, homodimers or heterodimers with primers. Specificity of probes was evaluated through NCBI BLAST and by running the primer-probe combinations on both strains and other available lactobacilli and oral bacteria.

**Table 1:** Sequences for primers and probes for the *L. reuteri* strains.

**Name**	**Sequence (5′-3′)**	**Source**
LDSM Forward	TTAAGGATGCAAACCCGAAC	Vestman et al. (Romani Vestman et al., 2013)
LDSM Reverse	CCTTGTCACCTGGAACCACT	Vestman et al. (Romani Vestman et al., 2013)
LDSM Probe	FAM-TTGGTAGGCTTGTTGAAAATCGCTA-Tamra	This study
LATCC Forward	GACAGTGGCTAAACGCCTTC	Vestman et al. (Romani Vestman et al., 2013)
LATCC Reverse	AATTCCACTTGCCATCTTCG	Vestman et al. (Romani Vestman et al., 2013)
LATCC probe	FAM-ATGACCTTGTAGGAGCCGTCTGGATAC-Tamra	This study

### Study design and participants

Prior to conducting the study, ethical approval was obtained from the Ethics Committee Research UZ/KU Leuven (reference: B3222025001672). The study was conducted in accordance with the ICH-GCP principles, the latest version of the Declaration of Helsinki, the Oviedo Convention on Human Rights and Biomedicine and applicable laws and regulations. 40 healthy participants participated in the day-night study and were randomized into the morning or night group through sealed envelope. As an exploratory colonization study rather than a confirmatory trial, no a priori power calculation was performed. Sample size was determined pragmatically by recruitment feasibility, and is comparable to or larger than previous oral probiotic colonization studies. 20 healthy participants participated in the colonization study. Both studies' participants were generally healthy and had no signs of oral diseases (see inclusion & exclusion criteria in supplementary information).

### Day-night study

Two groups of 20 participants each received a single lozenge, administered either in the morning or at night, randomization was performed through sealed envelopes with instructions for taking the probiotic either the morning or night. Both groups collected 2 mL of saliva before performing routine oral hygiene and then let the probiotic lozenge slowly dissolve on their tongue without mastication of the tablet (lozenges took up to 5 min to dissolve). A second 2 mL saliva sample was collected 8 h later, with food and liquids restricted during the final hour beforehand. The night group were instructed to brush their teeth approximately 10 min before bed and collect saliva in the morning shortly after waking up.

### Colonization study

Saliva was collected at baseline, days 7, 14, 21, and 28 during the 28 day probiotic administration period (twice daily, dissolution on the tongue without mastication). At day 28, two calibrated examiners sampled the tongue and palate (6 swipes with cotton swabs), supragingival plaque (6 teeth with a scaler), and interdental plaque (4 teeth with dental floss) in a standardized manner. All samples were taken 8 h after the morning dose, to avoid probiotics being present from said dose, as in most individuals probiotic from that dose is washed out (see day-night study). Saliva collection continued for the subsequent 7 day washout period.

Participants completed a questionnaire on oral hygiene habits: brushing frequency, toothbrush type, interdental cleaning, tongue scraping, toothpaste brand, and mouthrinse use.

### Sample collection, storage, processing, and microbial analysis

At each sampling point, 2 mL of unstimulated saliva was collected by drooling into Falcon tubes, while cotton swabs, interdental floss, and supragingival plaque were collected in 1 mL PBS. Samples were vortexed for 30 s, and the recovered liquid was immediately frozen at −20 °C until DNA extraction.

DNA was extracted using the QIAamp® DNA Mini Kit (QIAGEN, Hilden, Germany) and analysed with a CFX96 real-time PCR system (Bio-Rad, Hercules, CA, United States). Total bacteria were quantified with 16S rRNA gene primers (forward: P338:ACTCCTACGGGAGGCAGCAG; and reverse: P518:ATTACCGCGGCTGCTGG) with SYBR chemistry according to (Van Holm et al., 2021). *L. reuteri* quantities were evaluated with the species-specific primers and probes (Table 1) with probe chemistry according to (Van Holm et al., 2021).

15 µL of extracted DNA was sent for sequencing on an Illumina MiSeq platform (LGC, Berlin, Germany) of the 16S rRNA V3–4 region with the primers 341F (5′ – CCT ACG GGN GGC WGC AG – 3′) and 785Rmod (5′ – GAC TAC HVG GGT ATC TAA KCC – 3′) according to (Klindworth et al., 2013).

### Retrospective analysis: probiotics as adjunct after mechanical debridement of periodontitis patients

In another study (Saghi et al., publication in progress), periodontitis patients were prescribed Prodentis lozenges for 3 months either with a 2 weeks chlorhexidine regimen (group B; 22 patients) or without (group A; 24 patients), following mechanical debridement. According to the clinical protocol, microbial samples (saliva, plaque, and tongue swabs) were collected at baseline, 3- and 6 months for potential microbiome analysis to assess the effect of the probiotic use. More information on the study design can be found in the supplementary information.

In the current study, DNA from the samples were extracted from these samples and analysed for probiotic content, as described above, without performing full microbiome analysis. A secondary analysis was conducted focussing solely on the clinical parameters was performed and their association with concentrations of both *L. reuteri* strains in the microbial samples at 3 months.

### Statistical analysis

Statistical analysis for qPCR was performed in R 4.2.0 (https://cran.r-project.org/). Statistical differences for nonparametric data were analysed with a Kruskal-Wallis test with Dunn’s post hoc or an ANOVA with Tukey HSD for parametric data (95% CI). Pearson correlation was performed for linear correlations. Effect sizes and exact p- For pairwise comparisons, a Mann–Whitney U test (Wilcoxon rank-sum) was used, with Benjamini–Hochberg correction for multiple comparisons. Effect sizes were reported as epsilon-squared (Kruskal–Wallis), eta-squared (ANOVA), the rank-biserial correlation (pairwise Wilcoxon), Tukey mean differences, and Pearson's R (correlations), each with 95% confidence intervals. values for all comparisons are reported in Supplementary Tables 1–7.

Amplicon sequences were analysed using the DADA2 package in R (version 1.30.0) as described by Callahan et al. (Callahan et al., 2016). Primer sequences were removed, and reads were truncated based on quality scores (truncQ = 2). Reads with ambiguous bases or high expected errors (maxEE = 2,2) were filtered out. After dereplication, denoising was performed using the DADA algorithm with pooled sample inference (pooling = TRUE). Error rates were inspected and, upon validation, denoised reads were merged. Chimeras were removed, and the resulting ASV table was taxonomically classified using the Naive Bayesian Classifier with the SILVA v138.1 training set (Quast et al., 2013). The resulting read count table with taxonomic annotation was further processed in R (version 4.4.2). Rarefaction curves were used to assess if sufficient sequencing depth was obtained (vegan package version 2.6–10 (Oksanen et al., 2022); Supplementary 10). ASVs with a total abundance ≤1 across all samples were also removed.

Alpha diversity was calculated using the phyloseq package (version 1.50.0 (McMurdie and phyloseq, 2013)). PCoA based on Bray-Curtis dissimilarity was performed on the proportional community composition using the vegan package and statistical differences between groups were determined through permutational analysis of variance (PERMANOVA) with Holm correction for multiple comparisons (vegan package). Distance-based redundancy analysis (db-RDA) was conducted using Bray–Curtis dissimilarity to evaluate the influence of sampling location and retainer status on the proportional microbial community composition at the genus level. Analyses were performed using the vegan package, following the approach described by Minnebo *et al*. (Minnebo et al., 2023). Results were visualized as Type II scaling correlation triplots. A Holm correction was applied to account for multiple testing. Adjusted R² values, calculated using a subtractive procedure, are shown in the top-right corner of each db-RDA plot. The first two canonical axes are annotated with their respective constrained eigenvalues. Site scores represent weighted sums of genus scores, and factor levels of explanatory variables are displayed as centroids.

LEfSe analysis was performed on relative abundance data at timepoints 0 and 28 using the LEfSeR package (version 1.18.0 (Khleborodova et al., 2024)) to identify differentially abundant taxa between low and high retainers. The input data consisted of relative abundances, and the analysis was conducted using lefser() with Group as the class column. Visualization of the results was done using lefserPlot(), with customized colour schemes and trimmed feature names for clarity.

## Results

### Day/night study

To evaluate whether the time of day affects probiotic colonization, participants took a single probiotic lozenge either in the morning or at night. The amount of probiotic immediately after administration was measured by collecting saliva one minute after lozenge intake (Supplementary Figure 1). As expected, this initial amount was higher than at any later time point. Interestingly, the salivary production rate of individuals did not correlate with the probiotic’s concentration in saliva right after taking the lozenge.

**Fig. 1:**
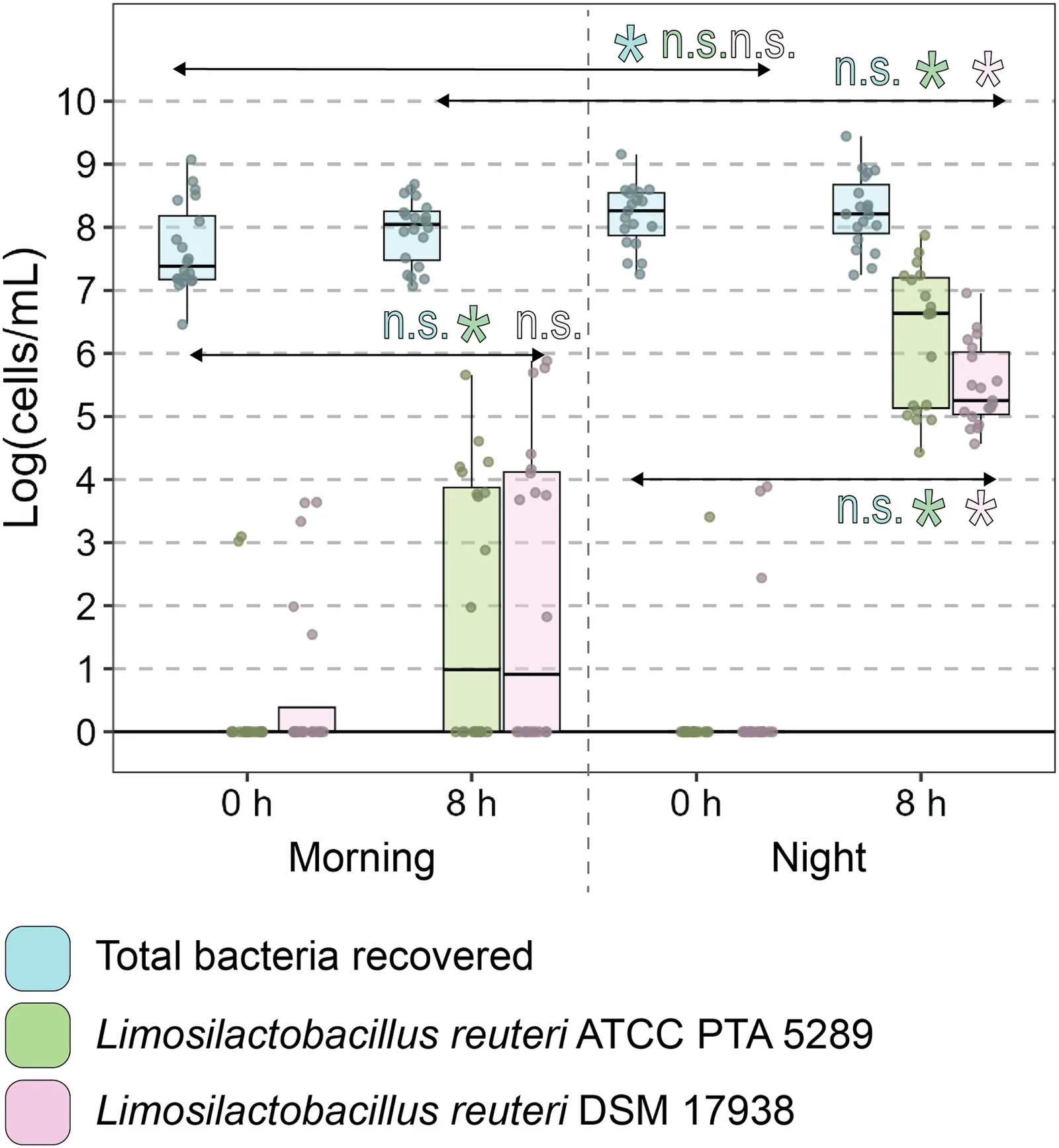
Bacterial detections in saliva after a single intake of probiotic lozenge for morning and night groups before and after the lozenge. Data are represented as single dots per participants of log cells per millilitre of qPCR detections of total recovered bacteria, *Limosilactobacillus reuteri* ATCC PTA 5289, and *Limosilactobacilllus reuteri* DSM 17938. Distributions per timepoint and group are presented per bacteria as bar charts. Significant differences between conditions are indicated with the asterisk in the representative colour of the bacteria above double sided arrows (n.s.: not significant; p < 0.05, Kruskal-Wallis with Dunn’s test; n = 20 per group, Supplementary Table 1).

After 8 h, probiotic levels in saliva had decreased (Fig. 1). In the morning group, only 10 participants still had detectable probiotics, resulting in a median of 1 log. In contrast, in the night group, all participants retained probiotics at significantly higher levels, with median values of 6.7 log and 5.2 log for LATCC and LDSM, respectively.

### Colonization study: salivary prevalence over time

Probiotic therapy typically involves repeated administration over an extended period, rather than a single dose. In this second study, participants received a probiotic lozenge twice daily for 28 days, followed by a 7-day washout period.

**Fig. 2:**
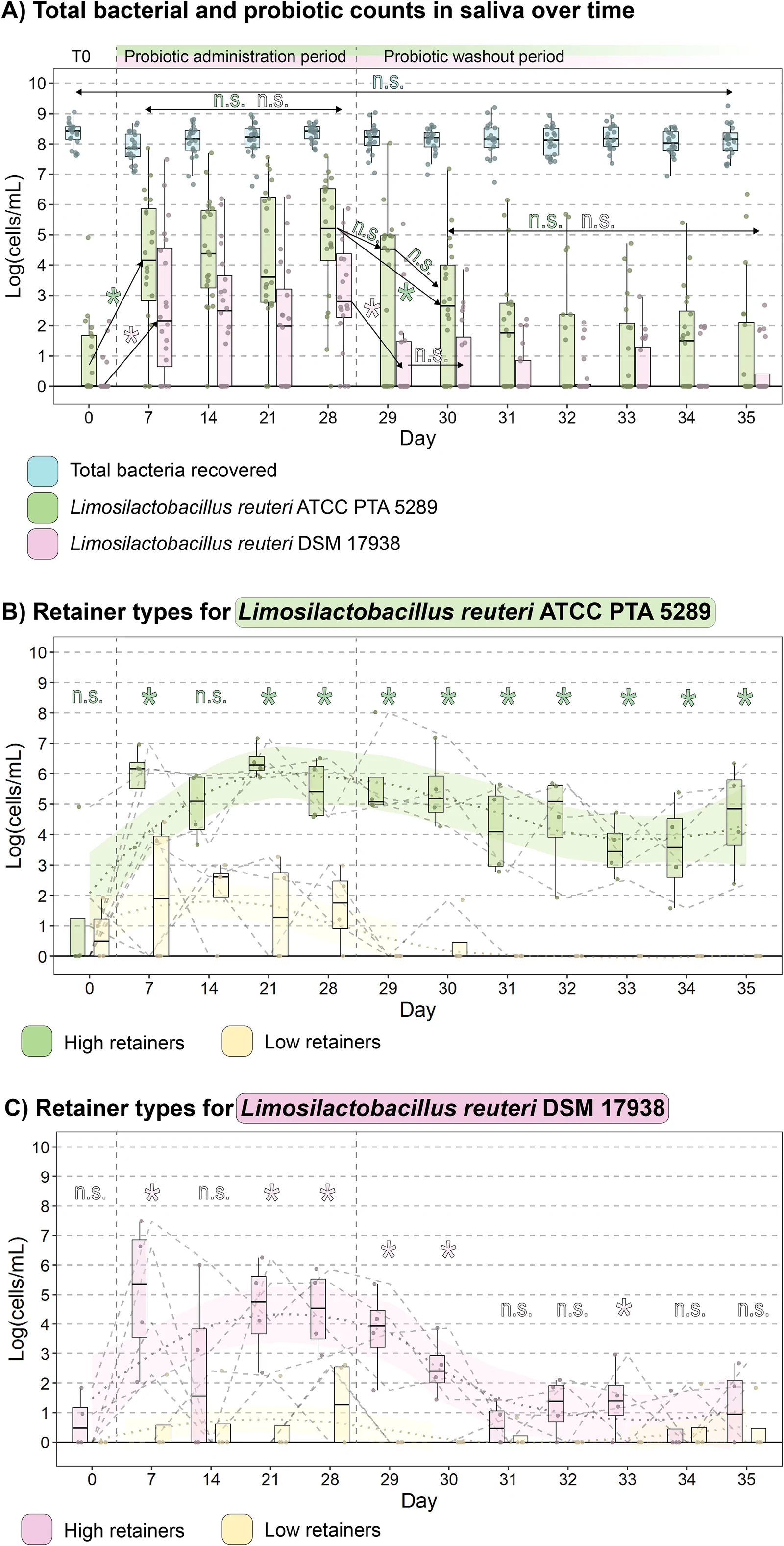
Evolution of probiotic presence in saliva over 28 days of twice daily probiotic intake and seven-day washout. **A:** Data are represented as single dots per participants of log cells per millilitre of qPCR detections of total recovered bacteria, *Limosilactobacillus reuteri* ATCC PTA 5289, and *L. reuteri* DSM 17938. Distributions per timepoint and group are presented per bacteria as bar charts. Significant differences between conditions are indicated with the asterisk in the representative colour of the bacteria above double sided arrows (n.s.: not significant; p < 0.05, Kruskal-Wallis with Dunn’s test; n = 20 per group, Supplementary Table 2–4). Only a selection of significant differences was presented to avoid chart cluttering. **B&C**: Comparison between high and low retainers for *L. reuteri* ATCC PTA 5289 and *L. reuteri* DSM 17938. Highest-and lowest 20% overall probiotic prevalence from Fig. 3A were subsetted. Significant differences between high and low retainers were presented with an asterisk per timepoint. (n.s.: not significant; p < 0.05, Kruskal-Wallis with Dunn’s test; n = 4 per retainer subset, Supplementary Table 5). Evolution of the prevalence per subject indicated with dashed lines.

After seven days of administration, the probiotics were detectable in the saliva of most participants, with no significant increase in concentration observed over the subsequent three weeks (Fig. 2A). Upon cessation of administration, LDSM levels declined significantly within one day, whereas LATCC required two days to show a significant decrease compared to levels at day 28.

After the 7-day washout period, the probiotics were not fully cleared from the oral cavity on average. However, rather than uniformly low levels across participants, some individuals retained disproportionately higher amounts of probiotic. Stratifying the cohort by selecting the top 20% and bottom 20% of retainers for both LATCC and LDSM revealed markedly divergent colonization profiles (Fig. 2B and C). 'High retainers' exhibited significantly higher probiotic levels during the administration phase compared to 'low retainers' at most timepoints, and notably, retained detectable levels of LATCC even after cessation of intake.

### Colonization study: site prevalence on day 28

**Fig. 3:**
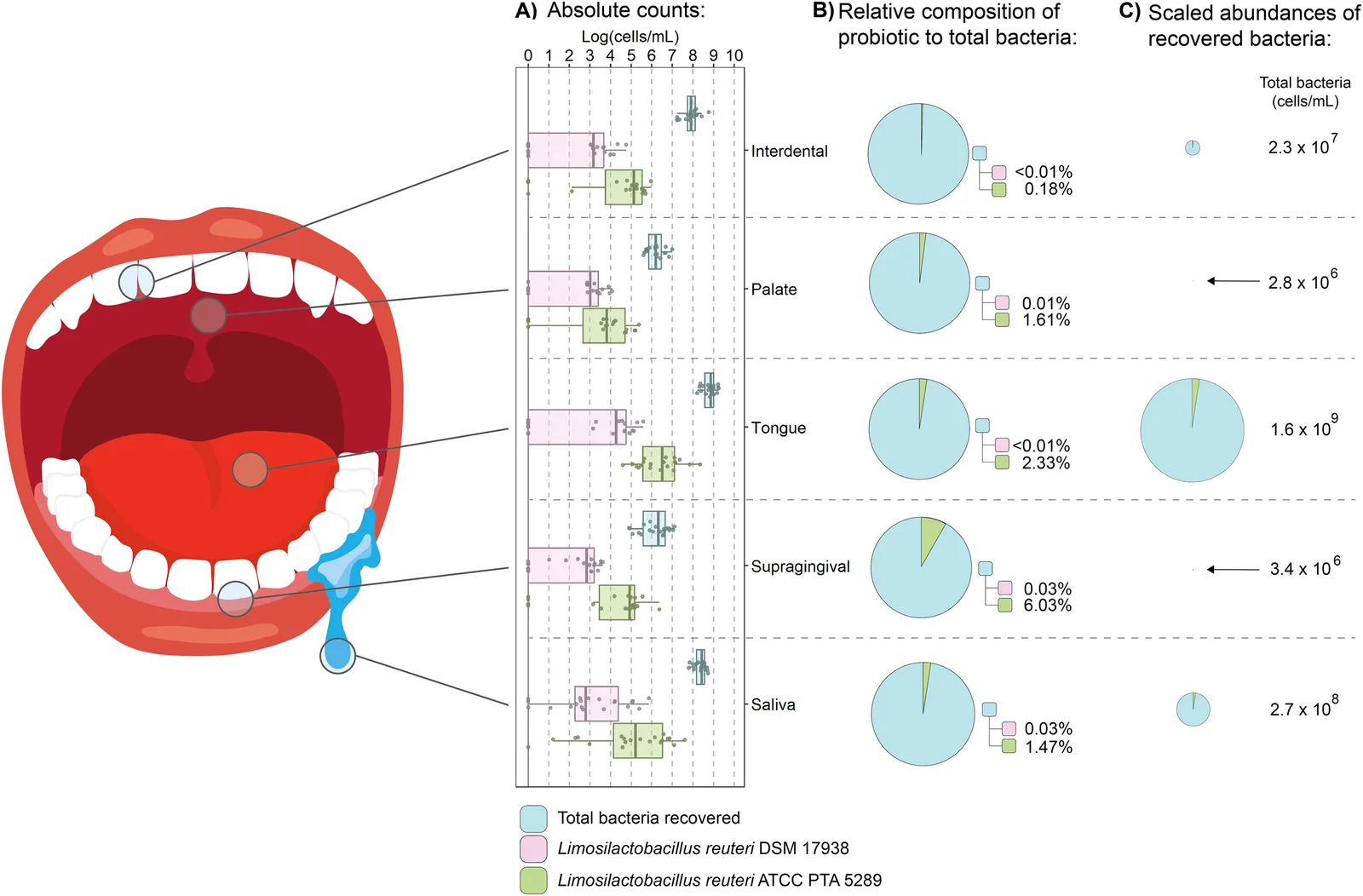
Prevalence of *L. reuteri* strains throughout the oral cavity after 28 days of twice daily probiotic intake. **A:** Absolute detections of bacteria from qPCR presented as log cells per millilitre per site. **B:** Relative proportion of the probiotic strains to the total detections of all bacteria. **C:** Scaled abundances of B) with total bacteria determining pie chart size.

To estimate the preferred adhesion sites of the probiotics, four additional oral niches were sampled on day 28. Both total bacterial load and *L. reuteri* strain abundance were quantified using qPCR (Fig. 3). The highest number of total bacteria was recovered from the tongue (1.6 × 10^9^ cells/mL), which also had the highest absolute amount of probiotic (1.91 × 10^7^ cells/mL; Fig. 3C). However, when considering relative abundance, the supragingival plaque showed the highest proportional presence of the probiotic, with LATCC comprising an average of 6.03% of the total biofilm (Fig. 3B).

The oral hygiene habits of participants were recorded to evaluate their effects on probiotic adhesion. Type of toothbrush, brushing frequency and smoking were discounted due to homogeneity and toothpaste type was discounted due to being too heterogenous. Interdental cleaning and use of a tongue scraper were evaluated, but did not significantly affect probiotic adhesion (**Supplemental figure 2**). Toothpaste brand use was too heterogeneous and no participants used mouthrinses.

**Fig. 4:**
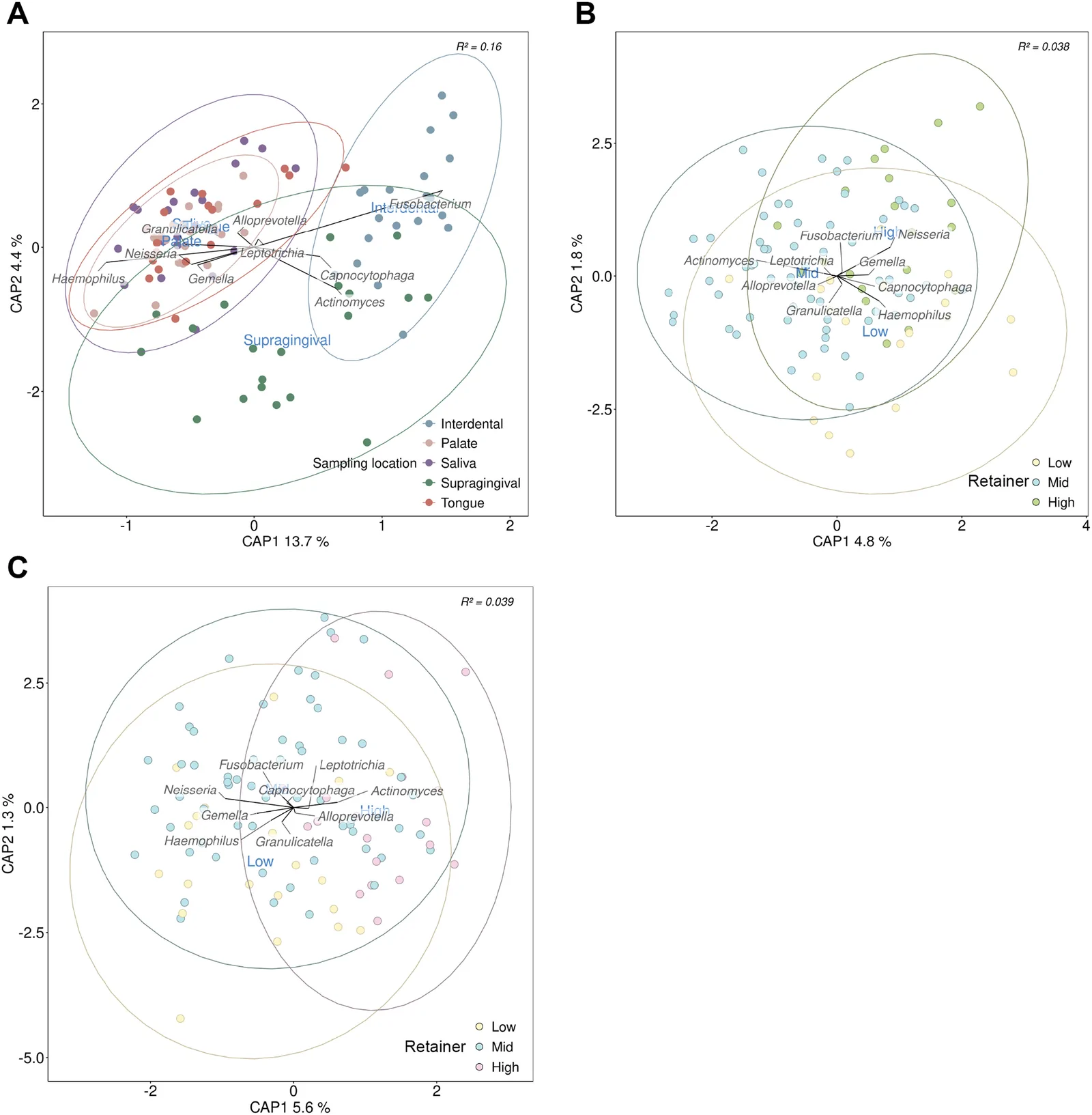
Distance-Based Redundancy Analysis (db-RDA) of samples on day 28. **A**: Data grouped per sample location, **B**: data grouped per retainer type for LATCC, **C**: data grouped per retainer type for LDSM. Ellipses are drawn per grouping (with a CI of 95%). Genera associated with each group are indicated by vectors (arrows) in the direction of the grouping. Length of the vector indicates strength of the association. R^2^ = proportion of variance explained. CAP = canonical analysis of principal coordinates.

**Fig. 5:**
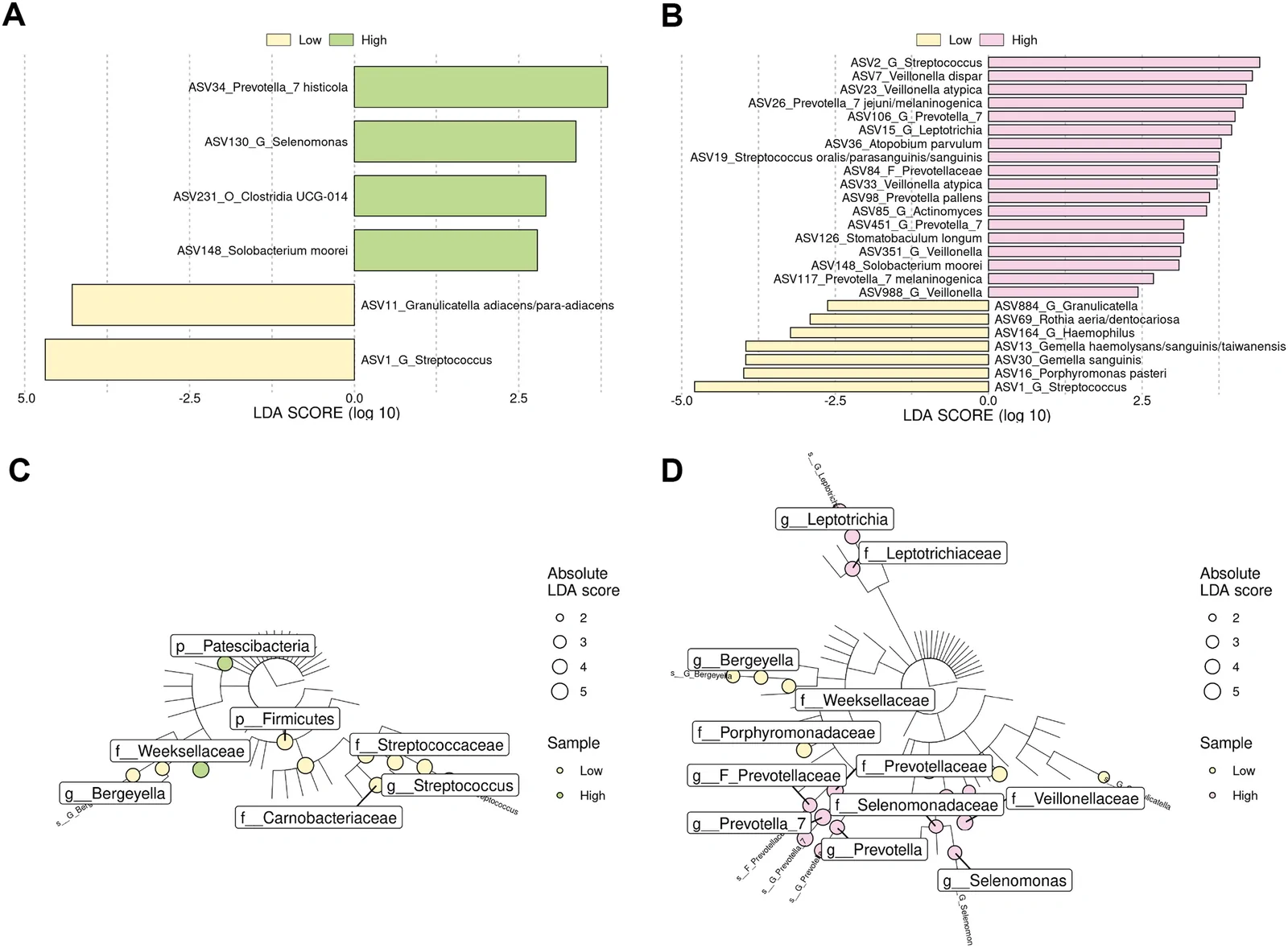
Bacteria associated with the *L. reuteri* strains in saliva on day 28. LEfSe of ASVs positively associated with the high and low retainers for LATCC (**A**) and LDSM (**B**). ASVs in green and pink are highly associated with either LATCC or LDSM respectively, making these potential bacterial biomarkers for the probiotic’s successful adhesion. ASVs in yellow are negatively associated with LATCC or LDSM, forming negative biomarkers/risk factors for successful adhesion of the probiotic. Cladograms indicate phylogenetic relationships of the bacteria positively (green/pink) or negatively associated (yellow) with either LATCC (**C**) or LDSM (**D**).

To evaluate if there is a link between the microbiome and the probiotic’s adhesion at the different sites, the samples of day 28 were also sequenced (Fig. 4, Fig. 5, **Supplementary Figures 3–7**).

Distance based redundancy analysis revealed that most of the dissimilarities between samples were linked with the sampling site (Fig. 4A: R^2^ = 0.16). Saliva, tongue and palate samples displayed large similarity and were dissimilar to supragingival and interdental plaque.

A low proportion of variance could be explained by the differences between retainer types (Fig. 4B and C; LATCC R² = 0.038 & LDSM R² = 0.039). However, principal component analysis (PCoA) showed significant differences between retainer types (**Supplementary figure 5**). There were no significant differences in the microbiomes between high and low retainers for LATCC, contrary to the difference in their retention of LATCC (Fig. 2B).

In the interest of identifying diagnostic markers in saliva to indicate whether an individual is a high or low retainer, the associations with certain bacterial ASV’s with the *L. reuteri* strains was examined with a LEfSe analysis (Fig. 5). *Prevotella* species were highly prevalent in high retainers for LATCC and LDSM, while *Heamophilus* and *Gemella* species were more prevalent in low retainers for both strains. Other than those genera, LATCC and LDSM differed substantially in associated ASVs with retainer types.

Most notable species for LATCC was *Prevotella histicola* in high retainers, while low retainers displayed more *Streptococcus* and *Granulicatella adiacens/para-adiacens.*

LDSM on the other hand had a lot more associated species, most notable of which were a *Streptococcus* (ASV2), multiple *Veillonella species* and a *Leptotrichia* in high retainers. Low retainers were associated with a different *Streptococcus* (ASV1), *Porphyromonas pasteri*, and multiple *Gemella* species. Other ASVs for the other sampling sites can be found in **Supplementary figure 6 & 7**. Notably is that the bacteria associated with the probiotics in saliva were also associated in the tongue and palate, while the supragingival- and interdental plaque only displayed some of these associations.

Contrary to the observable detections with qPCR, the lactobacilli were not very prevalent in the sequencing data (**Supplementary figure 3, 8 & 9**), The only notable lactobacillus was ASV44_G_HT002, which is described as an oral *Limosilactobacillus* isolate.

### Retrospective analysis: probiotics as adjunct after mechanical debridement of periodontitis patients

**Fig. 6:**
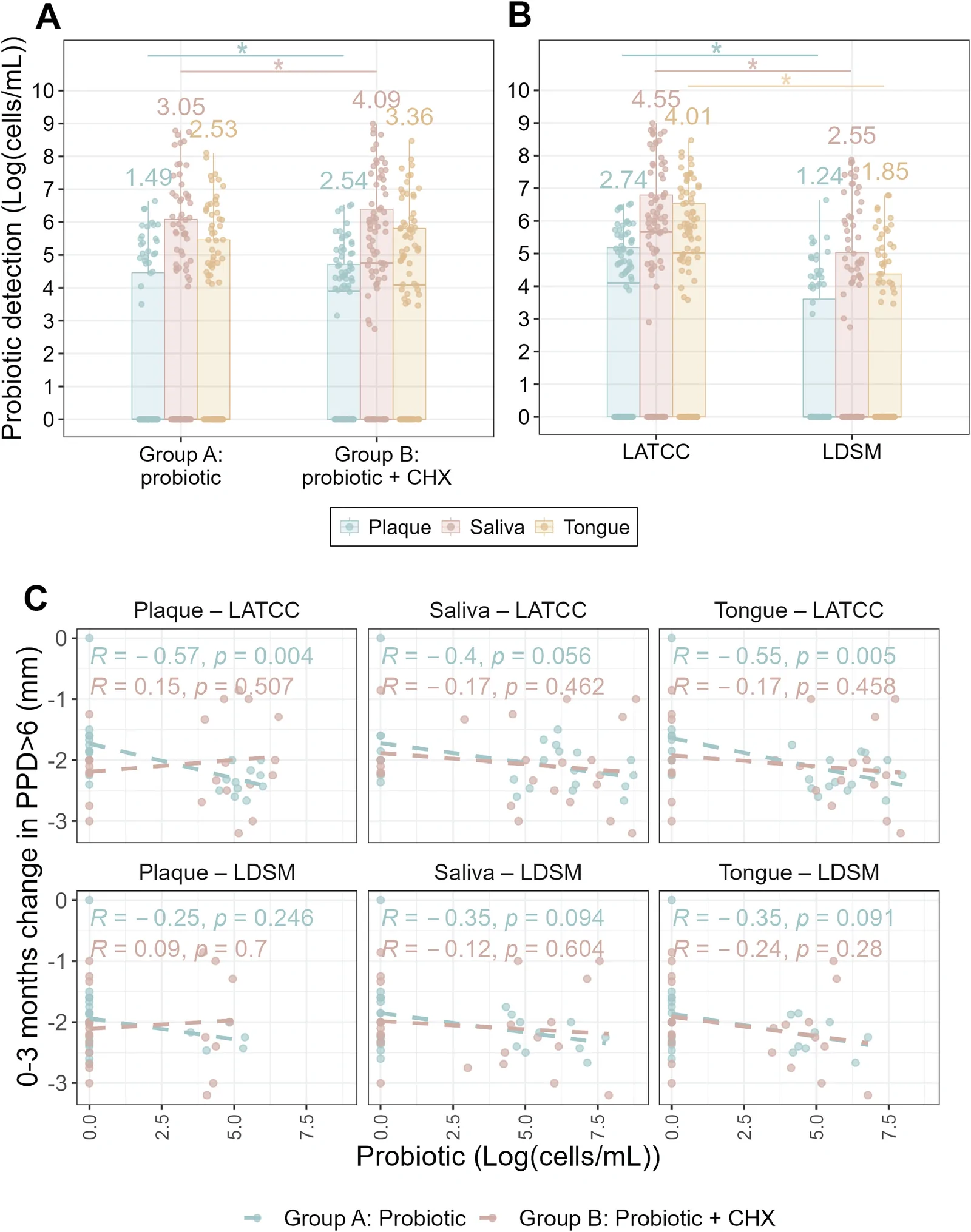
Retrospective analysis of probiotics as adjunct after mechanical debridement of periodontitis patients. Data presented are from a retrospective analysis of another study with 3 months of Prodentis probiotic lozenge with or without chlorhexidine mouthrinse (Saghi et al., publication in progress). **A:** Comparison of total probiotic presence per site between both groups. **B:** Comparison of prevalence of each strains per site (groups combined). Data of **A** & **B** are represented as single dots per participants of log cells per millilitre of qPCR detections of total recovered bacteria, *Limosilactobacillus reuteri* ATCC PTA 5289, and *Limosilactobacilllus reuteri* DSM 17938. Distributions per group are presented per sample type as bar charts with the arithmetic mean depicted in the representative colour. Significant differences between sample sites are indicated with the asterisk in the representative colour (n.s.: not significant; p < 0.05, Pairwise Wilcoxon tests with Benjamini-Hochberg correction Supplementary Table 6). **C:** Correlations of probiotic concentration and change in pocket probing depth of deep pockets (>6 mm). Data are expressed as the correlation of reductions in pocket probing depth in pockets deeper than 6 mm versus the log cells per millilitre of probiotics (*L. reuteri* ATCC PTA 5289, and *L. reuteri* DSM 17938). Data are facetted according per specific probiotic and per sample type (plaque, saliva and tongue swab). Correlations were assessed through Pearson’s correlation (95% CI, Supplementary Table 7). Other clinical parameters were also assessed, but showed no significant correlations.

To evaluate if these findings also translated to clinical results, a secondary analysis of data from another study was performed (Fig. 6). Patients receiving the probiotic and CHX (group B) had higher concentrations of total probiotics in saliva and plaque, but not on the tongue (Fig. 6A). LATCC was present in higher numbers than LDSM in all three sites (Fig. 6B). While several changes in clinical parameters were assessed (AL, Rec, FMBS, …; Saghi et al., publication in progress), only changes pocket probing depth (PPD) of deep pockets (>6 mm) yielded statistical significance (Fig. 6C). Higher presence of LATCC in plaque and on the tongue correlated significantly with a higher reduction in pocket depth after 3 months compared to baseline in the group that only received probiotics. This was not the case for LATCC in the saliva, and not for LDSM in any sample type. Interestingly, this effect was not present in the group that received the probiotic and CHX treatment (group B).

## Discussion

With the rise of probiotic therapy for oral health, there is a pertinent lack of understanding of where and how long a probiotic is present in the oral cavity. The current study presents a detailed look into how two commonly used *L. reuteri* strains adhere in the oral cavity.

In the first part, the daily timing of probiotic intake showed to have a major effect on how much probiotic is retained in the oral cavity over an eight-hour timeframe. Individuals taking the probiotic before bed had 100,000 times more probiotic still present in their saliva after 8 h than those that took it during the day.

In the second part of the study, a longer administration period of the lozenge twice a day for a month was performed. After 7 days, a consistent amount of probiotic was prevalent in the participants’ saliva, even though sampling was performed in the afternoon in which most non-adhered probiotic should be washed out as observed in the first part. While over 28 days the probiotic prevalence did not significantly change, after the administration ceased, both probiotics significantly reduced within 2 days and were barely present in most individuals after. However, this was not the case for all individuals. Especially for LATCC, some individuals even retained the probiotic for at least 7 days after administration in relatively high numbers.

A potential link between high and low retainers with the microbiome was also investigated, with some indication that bacteria of the genera *Prevotella, Streptococcus, Veillonella,* and *Haemophilus,* amongst others, are potentially involved with the adhesive success of the probiotic. However, more factors are likely at play.

### Day/night probiotic intake

The previously stated “100,000 times more probiotic in the night group” seems like an extremely large discrepancy between the groups, many individuals of the morning group did not have any detectable probiotic in their saliva. This is in stark contrast with the night group, in which every single individual still had detectable quantities, with a median of 5 log higher, resulting in the 100,000 times difference. The most likely explanation to the observed effect is the lower salivary flow during the night (Dawes, 1972), and lack of intake of food and drinks, resulting in lower washout than during the day.

Currently, the only recommendation for probiotic intake, backed by common sense rather than scientific proof, is administer probiotics after brushing and flossing, avoiding the immediate removal of the probiotic. As observed in the current study, at least one probiotic dose should be taken at night, ensuring prolonged presence of the probiotic.

### Probiotic colonization & localization

As previously stated, while there are many clinical studies on probiotics for oral diseases, most of these do not evaluate the content of probiotic in the oral cavity.

Of the studies that do evaluate oral adhesion, most relevant to the current study, Alforaidi et al. evaluated adhesion of LATCC and LDSM in probiotic drops for four weeks and a 5 week follow-up period in 13 individuals (Alforaidi et al., 2020). Similarly to the current study, one week after administration, most individuals lost most of the LATCC in their saliva. In surprising contrast, in their study, LDSM was present for longer than the LATCC strain, with two individuals harbouring the LDSM strain prior to the study without ever having taken the product previously.

While probiotics seem to typically disappear rather quickly, an interesting report by Yli-Knuuttila et al., had to exclude an adult individual due to them being permanently colonized by *L. rhamnosus* GG, likely acquired from a probiotic therapy at age 10 (Yli-Knuuttila et al., 2006). In this study, *L. rhamnosus* GG disappeared in most individuals within a week. Ravn et al. also describe this transient nature of several probiotics (Ravn et al., 2012). This might not be surprising as most probiotics, being lactobacilli, tend to form <1% of the normal oral microbiome (Badet and Thebaud, 2008; Caufield et al., 2015).

Rising in the field of gastrointestinal probiotics is the personalized selection of probiotics as “precision probiotics” (Veiga et al., 2020). This approach considers the individual, their pathology and which probiotic should be used in a bottom-up strategy. While this is not typically considered for oral probiotics, the current study demonstrated that several individuals responded significantly better or worse. The personalized selection of probiotics is likely also required for oral probiotics. Part of this personalization is the selection of suitable strains. As observed by Haukioja et al., even within the same species, in vitro ‘oral’ adhesive potential varied widely (Yli-Knuuttila et al., 2006). None of these strains were oral isolates, posing the question of how these would perform compared to the tested isolates. Of the two *L. reuteri* strains used in the current study, the LATCC always outperformed the LDSM strain. A potential explanation for this difference in efficacy can be attributed to that the LATCC strain was isolated from the oral cavity of a healthy Japanese woman, while the LDSM strain was isolated from the breast milk of a Peruvian mother (Liu et al., 2010). Despite being both *L. reuteri* strains, the LATCC strain is native to the oral cavity and likely possesses mechanisms making it more capable of remaining in the oral cavity. Although the observation that LDSM strain remained more persistent in the study by Alforaidi et al. (Alforaidi et al., 2020), contradicts this hypothesis. While administered daily dose is the same, in their study, the probiotic was administered as oil drops to Swedish participants, which may somehow favour the adhesion of LDSM. And even within orally derived samples, different isolates within the *L. reuteri* species show different effectivity against oral pathogens such as *Candida albicans* (Rajão et al., 2025).

However, poorer long-term adhesion does not exclude efficacy as the transient presence of the LDSM strain might be sufficient to exert its probiotics effects. Moving forward, selection of novel probiotics should consider oral isolates as extended prevalence as observed in the current study, or an evaluation of the novel probiotic’s oral adhesive capacities. Both approaches might allow the probiotic more time in the oral cavity to exert their beneficial effects, which is currently under investigated. While the probiotic might not be orally active, they may also exert a systemic effect by heading towards the gut.

To our knowledge only one study has investigated the localization of a probiotic in different sites of the oral cavity. In this study, Horz et al. evaluated the adhesion of *S. salivarius* K12 in a single individual after administration of 4 lozenges/day for 3 days (Horz et al., 2007). Most of the probiotic was detected on the pharynx, followed by the tongue. The *S. salivarius* strain was retained for up to 8 days with no detections at day 14. While *S. salivarius* K12 was isolated from saliva, its therapeutic potential for pharyngo-tonsillar infections, hint at an affinity for the pharynx (Gregori et al., 2016).

More recently, *S. salivarius* M18, also an oral isolate, delivered through a probiotic toothpaste, also showed persistence after discontinuation (Sali et al., 2025). However, their method of detecting *S. salivarius* M18 through inhibition of indicator strains instead of species specific methods does not exclude that other *Streptococcus* or native *S. salivarius* species were detected instead (Sali et al., 2025). Additionally, the timing of when saliva was collected was not mentioned, potentially risking the carryover of that days dose (Sali et al., 2025).

In the current study, the largest absolute number of *L. reuteri* was recovered from the tongue, likely due the direct contact of tongue and lozenge. Despite this, the largest proportional prevalence of *L. reuteri* was in the supragingival plaque, forming a substantial part of the biofilm. Once again, the overwhelming majority of the adhering *L. reuteri* was LATCC, the oral isolate, emphasizing the capabilities of an oral isolate.

### Probiotic-microbiome interactions

What differentiates the high and low retainers? If probiotic therapy is to be improved, properly identifying which individuals do and do not retain or respond well to certain probiotics may be crucial in improving probiotic therapy as a whole.

The oral microbiome is exceptionally resilient to outsiders (He et al., 2014; Zhou et al., 2013), but how this resilience relates to the colonization for probiotics has hitherto the current study yet to be investigated. Identifying which bacteria are positively or negatively associated with the *L. reuteri* strains may aid in the understanding of what is required or detrimental for their adhesion. To this end, the participants’ microbiomes were sequenced.

Some differences in their microbiomes between high and low retainers were observed, with certain genera and specific ASV’s associated with each. However, caution should be taken with extrapolating these results to a general population due to the limited size of the high and low retainer groups (each n = 4). While together they might give an indication whether an individual might respond well, a larger population is required to provide definitive biomarkers.

Nevertheless, preliminary candidate biomarkers to identify if a person is a high or low retainer could be obtained from the LEfSe analysis. Both *L. reuteri’s* successful incorporation are associated with *Veillonella-* and *Fusobacterium* species. Thus, they seemingly require classical bridging organisms. Curiously, high retainers for both *L. reuteri* strains had a high association with *Prevotella histicola*, a saccharolytic anaerobe (Downes et al., 2008), with seemingly no described interactions with probiotics. Perhaps more predictable, the well-known bridging organism, *Fusobacterium nucleatum* (Zhang et al., 2022), has recently been found to form profound interactions with both LATCC and LDSM in vitro (Yang et al., 2025), explaining the association observed in the current study.

Interestingly, low retainers to both *L. reuteri* strains had a higher LDA for *Rothia* (*aeria*), *Streptococcus* species, which are typically more health associated species (Mazurel et al., 2023; Wade, 2013). The niche for beneficial species might be already be fulfilled by these species, which either passively or actively exclude the *L. reuteri* strains.

While one *Streptococcus* species was negatively associated with both LATCC and LDSM (ASV1_G_Streptococcus), LDSM high retainers were also associated with another *Streptococcus* (ASV2_G_Streptococcus). Both, unfortunately, unclassified.

Interestingly, *Porphyromonas pasteri, Gemella-* and *Haemophilus* species were particularly enriched in low retainers. While their role in health is ambiguous or unknown, their negative association with *L. reuteri* probiotics might warrant further investigation.

While the of addition of *Limosilactobacillus* species should expectedly increase prevalence of *Lactobacilliaceae*, but this was not observed (**Supplemental figure 8 & 9**). In a previous study, we observed that species-specific qPCR detects higher numbers of the specific species than nonspecific techniques, likely due to the use of different primer sets and amplification protocols between the techniques (Mermans et al., 2024). Not a lot of other lactobacilli were detected, however one ASV did stand out. ASV44_G_HT002 correlated with the high retainers of LDSM (**Supplemental figure 7**), which is not all too surprising as the SILVA classifies *Limosilactobacillus* species to HT002 (Parente et al., 2022). HT002 is also identified as “an oral *Limosilactobacillus* sp. oral clone” (NCBI:txid242643). By sequencing the Prodentis® *L. reuteri* product for a different study (publication in process), both strains were also classified as G_HT002 with the same database.

While the microbiome is likely a crucial factor in the retention of the probiotic, there are likely other factors involved. A well-known factor in the colonization of gastrointestinal probiotics is their host’s diet (Chen et al., 2025). In a study on oral microbiome transplants, the role of diet was found to contribute around 12% of variance in microbiomes (Nath et al., 2025). The broader influence of food composition on oral microbiota homeostasis has also recently been reviewed (Chen et al., 2026), further underscoring diet as a likely modulator of probiotic adhesion, and thus treatment success, which warrants dedicated investigation. While largely unexplored for oral probiotics, some emerging evidence exists. For example, the presence of polyphenols in wine might improve the adhesion and functioning of *Streptococcus dentisani* 7746 (Esteban-Fernández et al., 2018). For oral probiotic foods, the food matrix is speculated being important for delivery and adhesive potential of the probiotic (Chua et al., 2020), such as the properties of cheese matrixes that contain lactic acid bacteria affecting their persistence in the oral cavity of rats (Ibarlucea-Jerez et al., 2024), although most current studies do not consider other confounding factors (Chen et al., 2026). The differences in adhesive capacity due to delivery (oil drops versus lozenges) or participants’ diet might also explain the differences in adhesion of the same *L. reuteri* strains between Alforaidi et al. and the current study (Alforaidi et al., 2020). While the Swedish and Belgian diets are likely not too dissimilar, both being Western European patterns rich in dairy and refined carbohydrates, the effects of more divergent global dietary patterns on probiotic incorporation could be considerably more pronounced.

Unfortunately, the effect of the delivery system is yet to be extensively investigated in humans. Probiotic foods may be beneficial for the delivery since active probiotics have better adhesive capabilities than inactivated probiotics typically found in lozenges (Van Holm et al., 2023).

This suggested use of oral probiotic foods might also pose the question to if lozenges are the best delivery method for oral probiotics. While having a lot of probiotic on the tongue might be beneficial for halitosis, other delivery methods, supplying the probiotic directly to the teeth and gingival margin, may be more interesting for caries and periodontitis.

Another of the host’s habits, their dental hygiene was speculated to affect the probiotic’s adhesion to the maintained site. Contrary to our hypothesis, oral hygiene habits did not significantly affect probiotic adhesion (**Supplemental figure 2**), although the sample size is rather small to conclusively exclude the effects of oral hygiene on probiotic adhesion.

Aside from the microbiome and the host’s habits, innate factors of the host might also affect the probiotic’s retention. Aside from salivary washout, saliva itself contains many factors meant to exclude exogenous bacteria (Amerongen and Veerman, 2002), including probiotics. Factors like salivary immunoglobins can display significant interindividual differences due to immutable factors (Brandtzaeg, 2007). Currently there is no evidence on if these affect oral retention of probiotics.

### Clinical implications?

While numerous RCTs and meta-analyses studies evaluate of probiotics versus a placebo on clinical outcomes, woefully few studies actually access presence versus absence of the probiotic and even less enumerate probiotic content within the microbiological samples. Even though a patient might have received the probiotic, as observed in the current study, it might be barely present in the highly dynamic oral cavity. Despite this, the continuous nature of several clinical outcomes leans to them being easily correlated with the actual concentration of probiotics in the oral cavity, which should be assessed in future clinical studies to differentiate between low and high retainers and their clinical outcomes.

While not originally designed for this analysis, the microbiological samples and clinical parameters from a study by Saghi et al. (publication in progress) were analyzed. Additionally, the use of an antiseptic in conjunction with the probiotic increased the overall retention of the probiotic, confirming their combined use as previously hypothesized (Lauwens et al., 2026). However, this increased probiotic presence in group B did not yet yield clinical results as the only significant effects were observed the probiotic only group, group A. The clinical benefits of CHX containing mouthrinse potentially masks some of the probiotics’ effects. While most clinical parameters did not yield significant effects, in the sites where the most improvement was possible, i.e. the deepest pockets, the presence of the orally adapted probiotic resulted in a better clinical outcome.

### Limitations

Only two probiotic strains were investigated, and their efficacy differed even within the species, suggesting broader variation across other probiotics. Future research should explore probiotic-host compatibility and the influence of diet and host factors on adhesion.

The 7 day follow-up was insufficient to determine if the high retainers maintained the probiotic longer, and longer administration and follow-up studies are required. Caution is required when interpreting the results for the high and low retainers as each group comprised only 4 individuals. These preliminary candidate biomarkers might not extrapolate to the larger population and require thorough validation before they can be used for such a purpose.

A noticeable discrepancy was observed between the qPCR- and sequencing results in the prevalence of the *L. reuteri* strains in proportion to the rest of the microbiome. Whether the probiotics do take up to 6% of the supragingival plaque (qPCR) or are barely present (16S rRNA gene amplicon sequencing). This most likely reflects several methodological factors rather than a true biological contradiction. First, the species specific designed primers for LATCC and LDSM confer greater sensitivity than the universal primers. Targeted methods were previously also observed to give higher numbers than non-specific community techniques (Mermans et al., 2024). Second, universal 16S amplification is prone to primer bias against low-abundance taxa, which are readily outcompeted during amplicon PCR. Third, strains were not truly absent from the sequencing data, but possibly collapsed into other low abundance taxa, such as the discussed G_HT002. Due to these limitations, the proportional estimates from the species specific qPCR were regarded as more reliable quantification.

While higher prevalence of probiotic can potentially be linked with better clinical outcomes, the retrospective analysis was underpowered to yield any conclusive results. Studies adequately powered to link probiotic prevalence with clinical outcomes are required to verify this observation.

## Conclusions

In summary, daily timing, the host, microbiome suppression and the origin of the probiotic strain all play a role in their colonisation of the oral cavity. However, these do not fully explain the clinical differences in response to the probiotics.

Better understanding the mechanisms behind this yet unknown retention can lead to a precision probiotic approach in which the best ‘probiotic fit’ is found on a patient-to-patient basis.

While the microbiome could form a predictive element in deciding probiotic fit, other host-related factors such as diet, healthcare habits, and immutable factors, warrant further investigation to obtain a full picture on interindividual differences in probiotic efficacy.

## CRediT authorship contribution statement

**Wannes Van Holm:** Conceptualization, Methodology, Validation, Formal analysis, Investigation, Data curation, Writing – original draft, Writing – review & editing, Visualization, Supervision. **Julie Marynissen:** Validation, Formal analysis, Investigation, Writing – original draft. **Lien Van Campenhout:** Validation, Formal analysis, Investigation, Writing – original draft. **Yorick Minnebo:** Formal analysis, Investigation, Data curation, Writing – review & editing, Visualization. **Fabian Mermans:** Formal analysis, Investigation, Data curation, Writing – review & editing. **Katalina Lauwens:** Methodology, Writing – review & editing. **Kobe Teughels:** Methodology, Validation, Investigation, Writing – review & editing. **Mehraveh Saghi:** Methodology, Validation, Writing – review & editing. **Naiera Zayed:** Methodology, Validation, Writing – review & editing. **Nico Boon:** Methodology, Writing – review & editing, Supervision. **Wim Teughels:** Conceptualization, Methodology, Writing – review & editing, Supervision.

## Declaration of competing interest

The authors declare the following financial interests/personal relationships which may be considered as potential competing interests:

Wim Teughels reports equipment, drugs, or supplies was provided by BioGaia AB. Wim Teughels reports a relationship with BioGaia AB that includes: funding grants and speaking and lecture fees. If there are other authors, they declare that they have no known competing financial interests or personal relationships that could have appeared to influence the work reported in this paper.

## Appendix Supplementary materials

Image, application 1
