# Supplementary material for: Oral colonization of probiotics: one size fits all?
Source: Curr Res Microb Sci. 2026 Jul 7;11:100642. doi: 10.1016/j.crmicr.2026.100642 (PMC13382399; doi:10.1016/j.crmicr.2026.100642)
Supplement: Supplementary file 1 [file mmc1.docx]

# Supplementary information: Oral colonization of probiotics: one size fits all?

**In- and exclusion criteria for the day/night and colonization study**

**Inclusion criteria:**

1. Voluntary written informed consent of the participant has been obtained prior to any screening procedures
2. 18 years of age

**Exclusion criteria:**

1. Participants eligible for this Trial must not meet any of the following criteria:
2. Any disorder, which in the Investigator’s opinion might jeopardise the participant’s safety or compliance with the protocol.
3. Any prior or concomitant treatment(s) that might jeopardise the participant’s safety or that would compromise the integrity of the Trial.
4. Participation in an interventional Trial with an investigational medicinal product (IMP) or device.
5. Participation in any other clinical study.
6. Patients who took systemic antibiotics up to 3 months prior to study visit #1.

**Study design of the retrospective study: Saghi et al. (publication in progress)**

The analysis in Figure 6 is a secondary analysis of samples and clinical data from a completed randomized controlled trial. Its design is summarised here to aid interpretation of Figure 6.

**Design and participants:** The source study was a 24-week, single-centre, double-blind, three-arm, placebo-controlled randomized controlled trial conducted at the Department of Periodontology, Çukurova University (Adana, Turkey; ethics approval 150/5, 01.07.2021; ClinicalTrials.gov NCT05548361; CONSORT-compliant).

Seventy-seven patients with generalized periodontitis stage III or IV, grade B, with at least three natural teeth per quadrant, were enrolled. Key exclusion criteria were localized or stage I–II periodontitis, grade A or C, systemic disease (e.g. diabetes), medications affecting the periodontium, antibiotic use within the preceding 3 months, pregnancy or lactation, and tobacco use. Randomization was performed by an independent investigator (via www.randomizer.org); study products were dispensed in identical, opaque, sequentially numbered bottles, and both participants and examiners were blinded.

**Interventions:** All participants received full-mouth scaling and root planing (SRP) with a full-mouth chlorhexidine (CHX) disinfection protocol. The three arms were: (i) probiotic (*Limosilactobacillus reuteri* DSM 17938 and *L. reuteri* ATCC PTA 5289; GUM Periobalance (no CHX), Sunstar) twice daily for 3 months plus placebo mouthrinses for 2 weeks; (ii) CHX (0.12%, GUM Paroex, Sunstar) twice daily for 2 weeks plus probiotic lozenge for 3 months; and (iii) CHX for 2 weeks plus placebo lozenges for 3 months.

Lozenges were dissolved on the tongue without mastication after oral hygiene routines and at least 30 minutes after mouthrinse rinsing. No other antimicrobial agents or mouthrinses were permitted.

**Outcomes:** The primary outcome was probing pocket depth (PPD; six sites per tooth, Merritt-B probe, calibrated examiner).

Secondary outcomes included: gingival recession (REC), clinical attachment level (CAL = PPD + REC), full-mouth bleeding score (FMBS), and full-mouth plaque score (FMPS).

The source trial was powered for its primary clinical endpoint (G*Power; 69 participants for 95% power, alpha = 0.01, effect size f = 0.25, repeated-measures ANOVA), and was therefore adequately powered for the clinical outcomes on which the present secondary analysis draws.

**Use for the current study**: Only the two probiotic-containing arms were included here, corresponding to Group A (probiotic) and Group B (probiotic + CHX); the CHX-only arm was excluded as it contained no probiotic to quantify in the patients. For each participant, strain-specific probiotic detection (qPCR for LATCC and LDSM in plaque, saliva, and tongue, as described in the methods from the other two studies) at the 3-month timepoint was related to the 0 to 3 month change in the number of deep pockets (PPD >6mm), as shown in Figure 6.

# Supplemental figures:


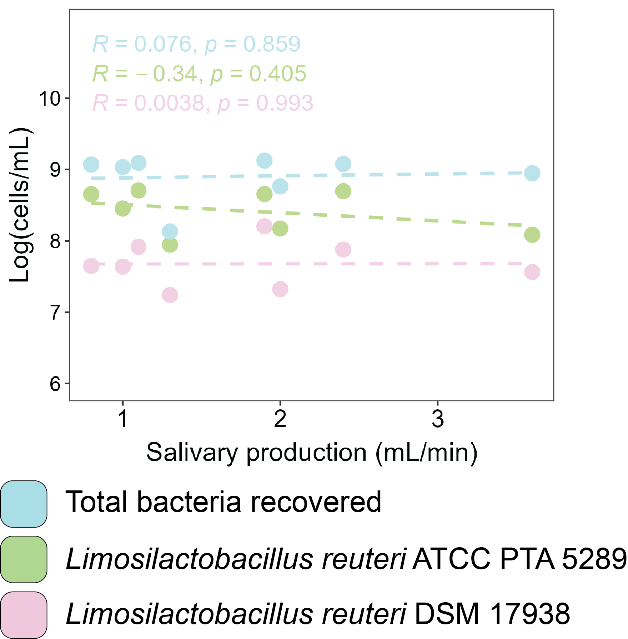


**Supplementary figure 1**: Total bacteria and probiotic prevalence right after taking a single lozenge in relation to the total amount of saliva produced over one minute.

After the lozenge was completely dissolved, participants were instructed to swallow all saliva and then collect all saliva produced in one minute and the salivary volume was recorded. Bacterial quantities from qPCR are expressed as the base ten logarithm of cells detected per milliliter of saliva. Pearson correlation was performed to estimate bacteria correlation to salivary production (n = 9).


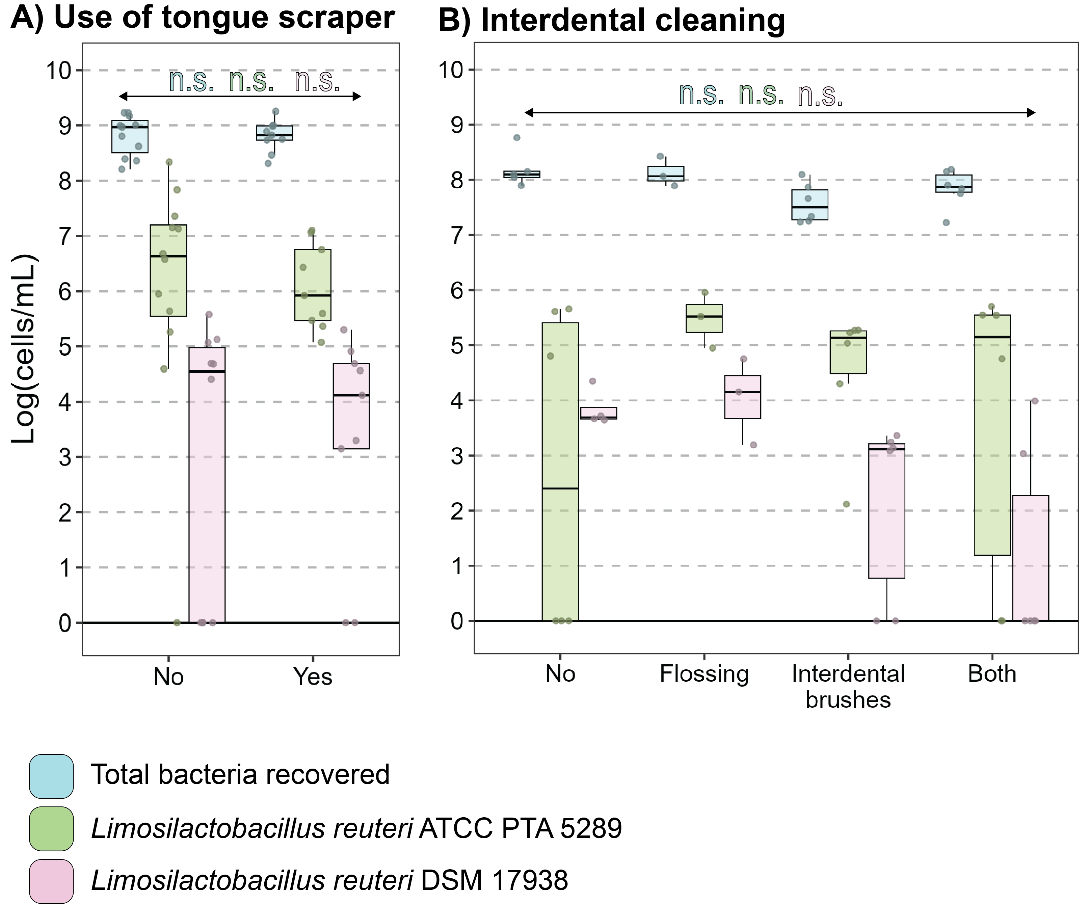


**Supplementary figure 2**: The effects of oral hygiene on probiotics in the cleaned sites sampled on day 28.

Data are represented as single dots per participants of log cells per milliliter of qPCR detections of total recovered bacteria, Limosilactobacillus reuteri ATCC PTA 5289, and Limosilactobacillus reuteri DSM 17938. Distributions per group are presented per bacteria as bar charts. The 20 participants were divided into respective groups according to their reported habits. No significant differences were observed between conditions as indicated with n.s.: not significant (p<0.05, Kruskal-Wallis with Dunn’s test).

**A:** Effect of tongue cleaner use on bacterial composition and number of detectable probiotics on the tongue.

**B:** Effect of interdental cleaning on bacterial composition and number of detectable probiotics in the interdental plaque.


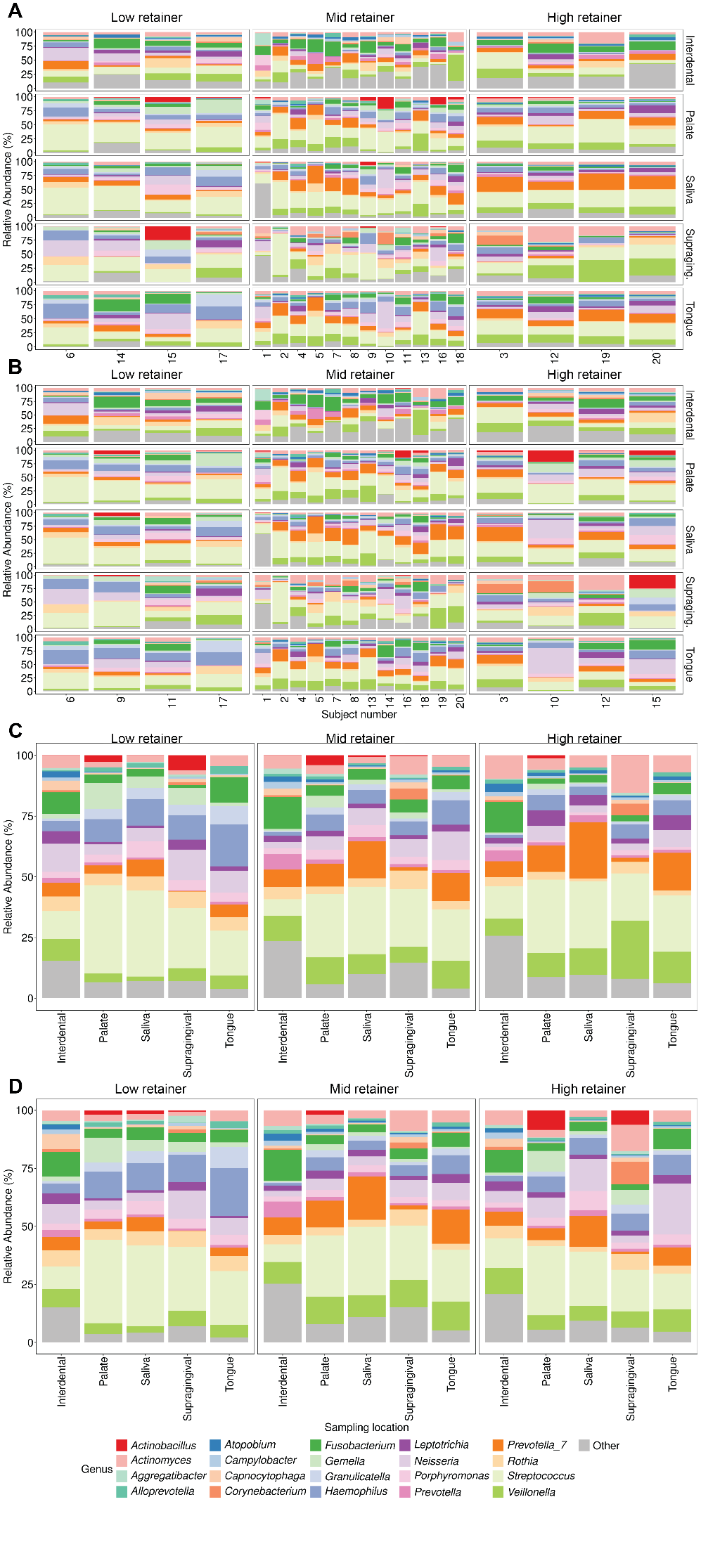


**Supplementary figure 3**: Relative abundances of bacterial genera in samples per retainer type for the *L. reuteri* strains on day 28, based on 16S rRNA gene amplicon sequencing.

**A:** Per subject for LATCC. **B:** Per subject for LDSM. **C:** Averaged for LATCC. **D:** Averaged for LDSM. Average relative abundances were calculated by grouping samples by sampling location, timepoint, taxon, and response, and computing the mean abundance within each group.


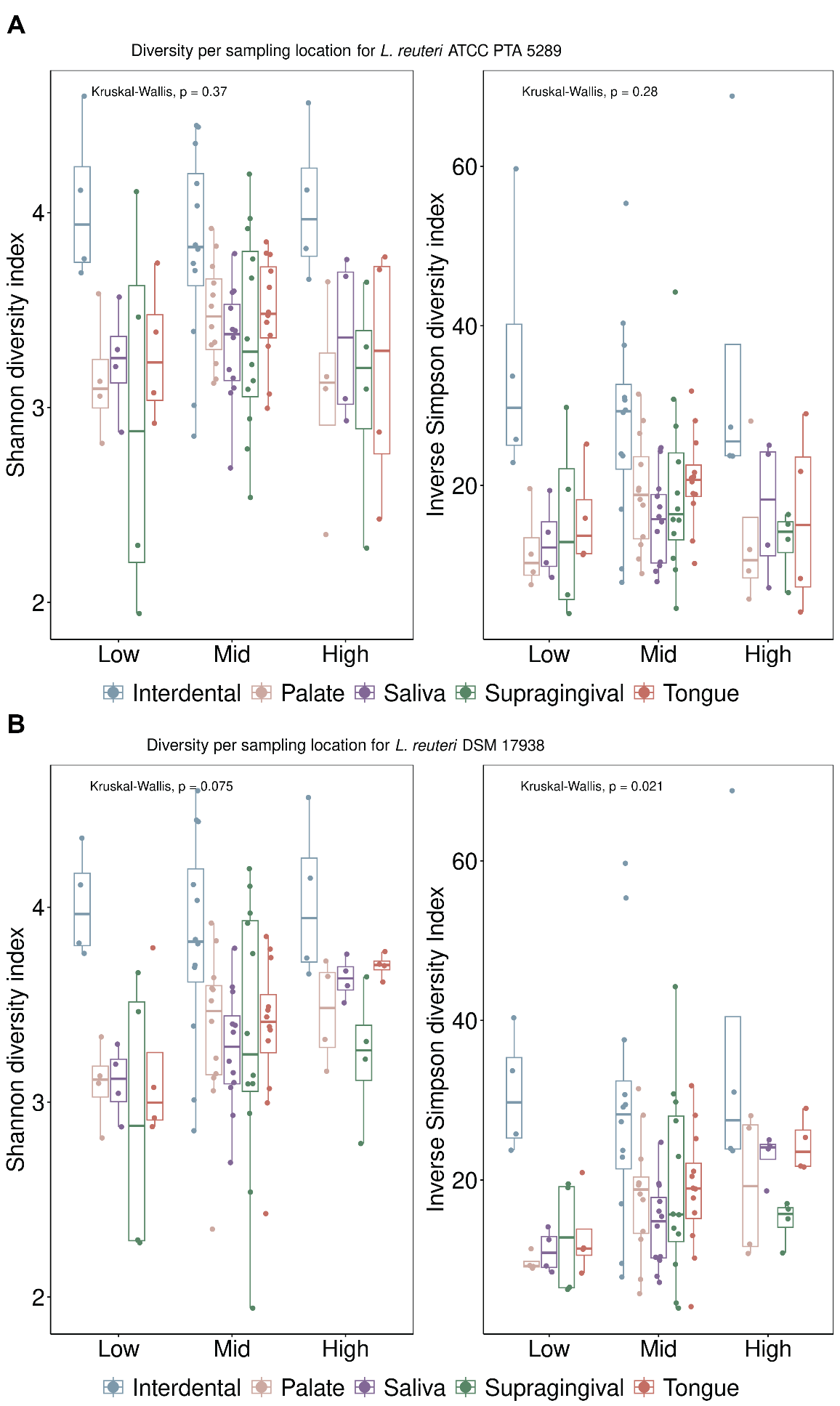


**Supplementary figure 4**: Alpha diversity (Shannon diversity and inverse Simpson diversity) per site per retainer type for the *L. reuteri* strains on day 28.

**A**: Alpha diversity grouped for LATCC. **B**: Alpha diversity grouped for LDSM.


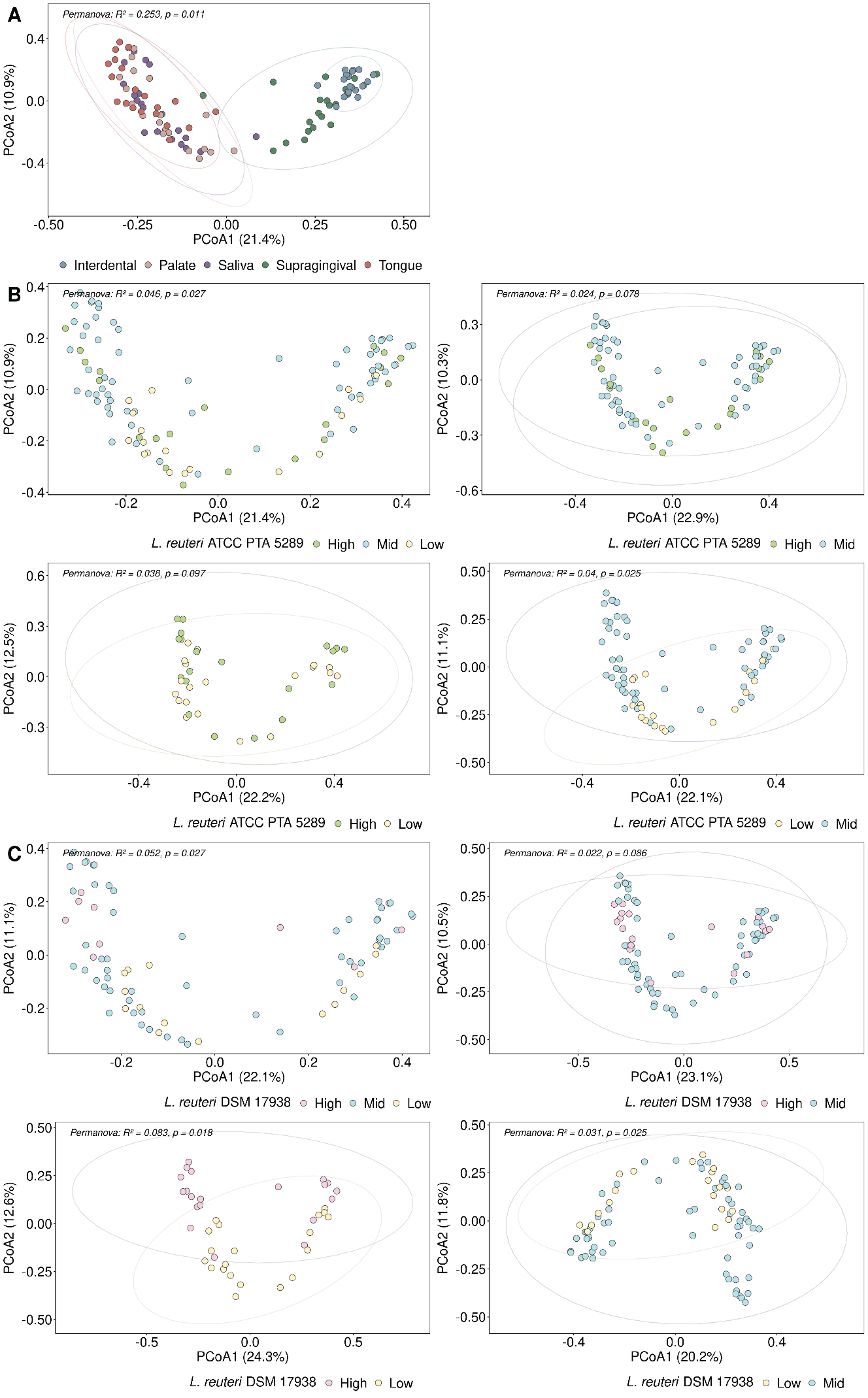
**Supplementary figure 5**: Principal Coordinates Analysis (PCoA) based on Bray-Curtis dissimilarity (beta diversity) of all samples collected on day 28. PERMANOVA analyses with Holm correction were performed with the corresponding R² and p-values shown in the top-left corner of each panel.

**A:** Beta diversity across all five sampling sites.

**B:** Beta diversity between retainer types for *L. reuteri* ATCC PTA 5289. The top-left panel includes all retainer types, while the remaining panels show pairwise comparisons between high, mid, and low retainers.

**C:** Beta diversity between retainer types for L. reuteri DSM 17938. The top-left panel includes all retainer types, while the remaining panels show pairwise comparisons between high, mid, and


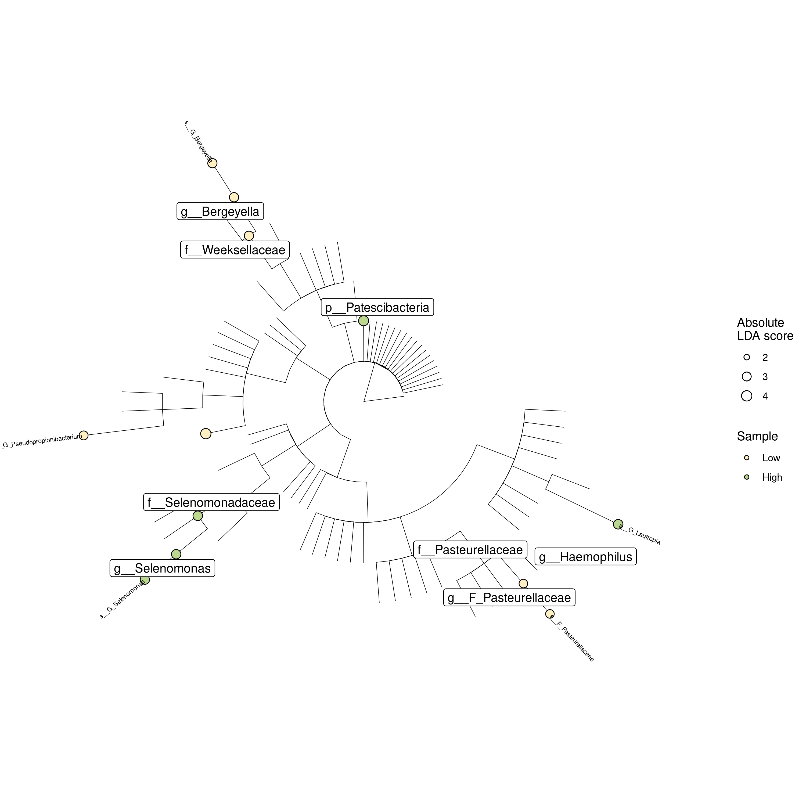

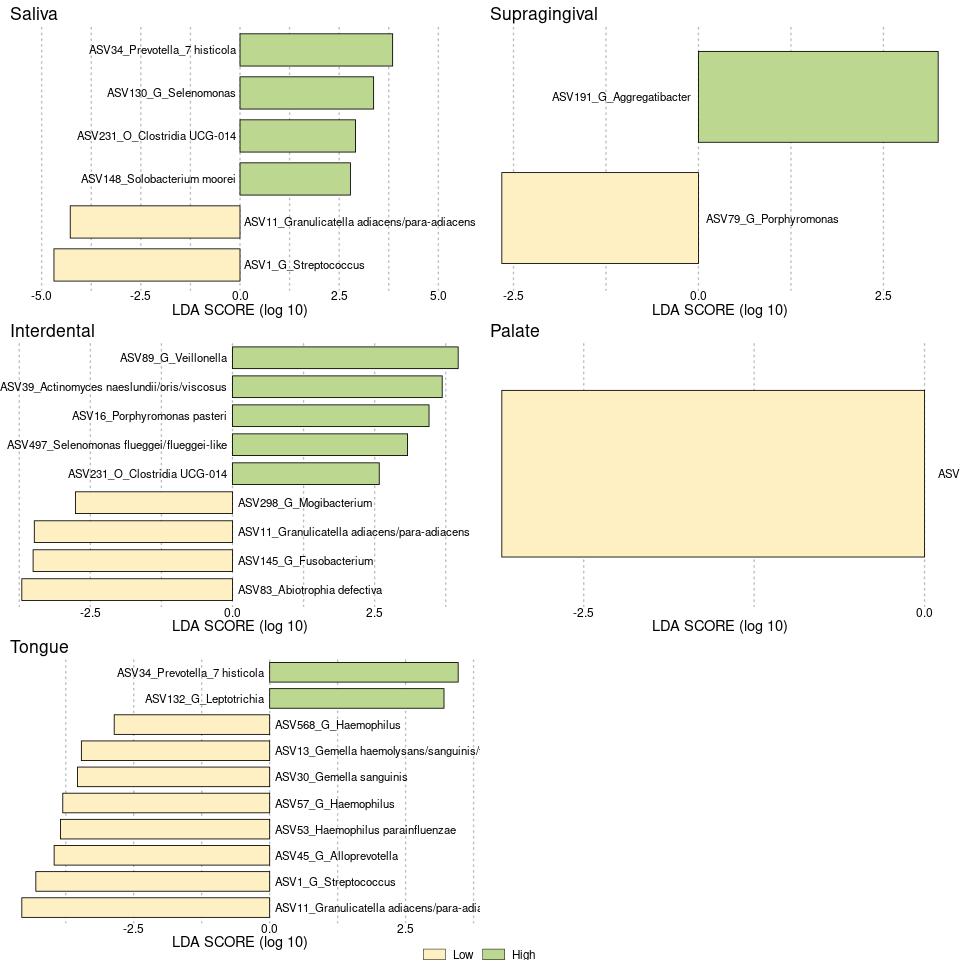
**Supplementary figure 6**: LEfSe & cladogram of all sampling sites for high and low retainers for LATCC on day 28. ASVs in green are highly associated with LATCC, making these potential bacterial biomarkers for the probiotic’s successful adhesion. ASVs in yellow are negatively associated with LATCC, forming negative biomarkers/risk factors for successful adhesion of the probiotic. The cladogram indicates phylogenetic relationships of the bacteria positively (green) or negatively associated (yellow).


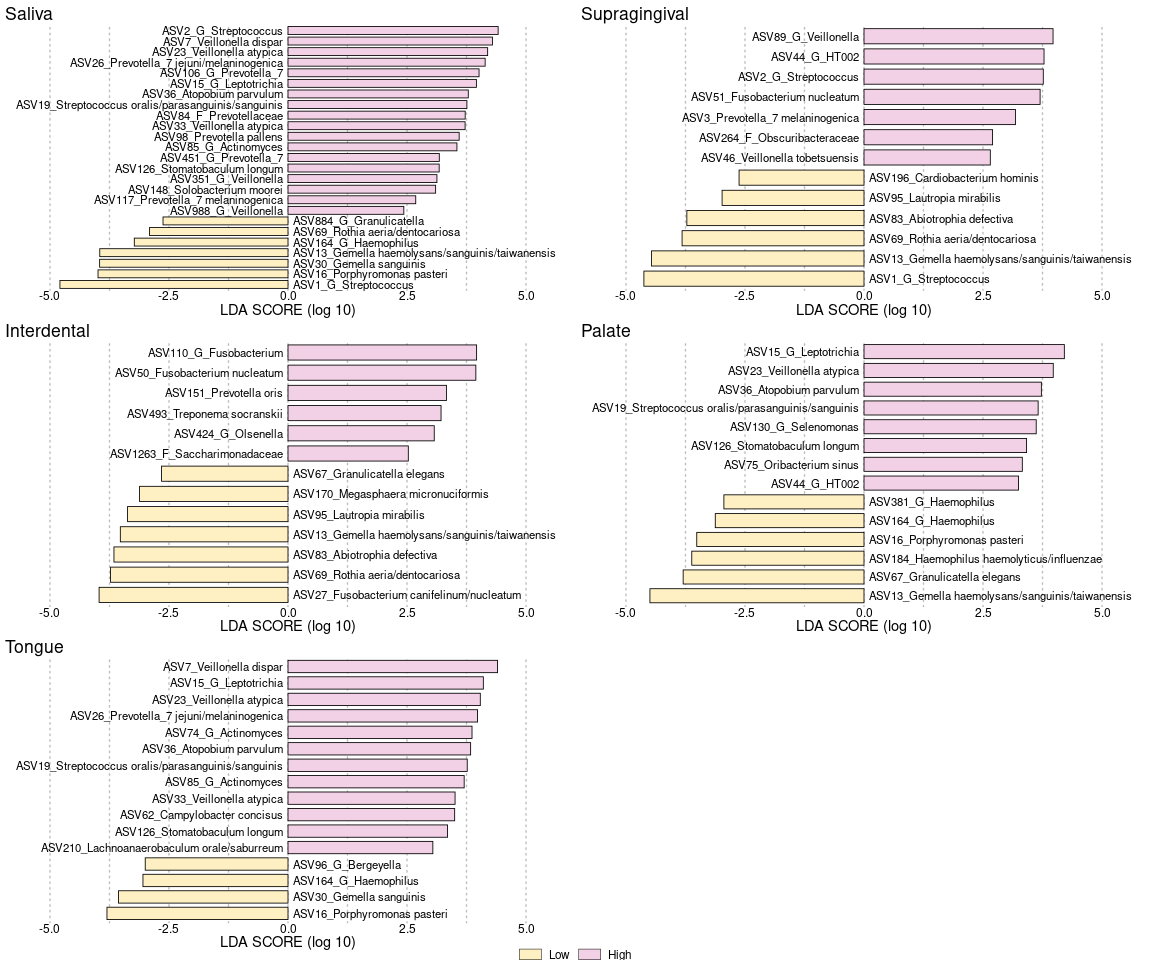


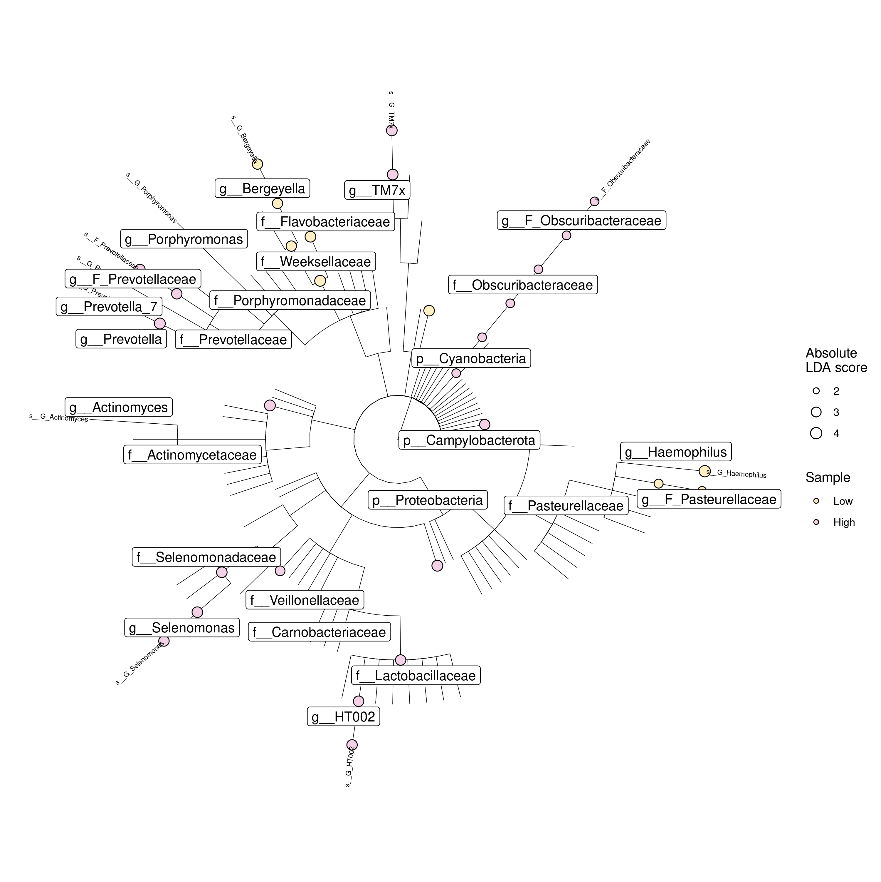
**Supplementary figure 7**: LEfSe & cladogram of all sampling sites for high and low retainers for LDSM on day 28. ASVs in pink are highly associated with LDSM, making these potential bacterial biomarkers for the probiotic’s successful adhesion. ASVs in yellow are negatively associated.
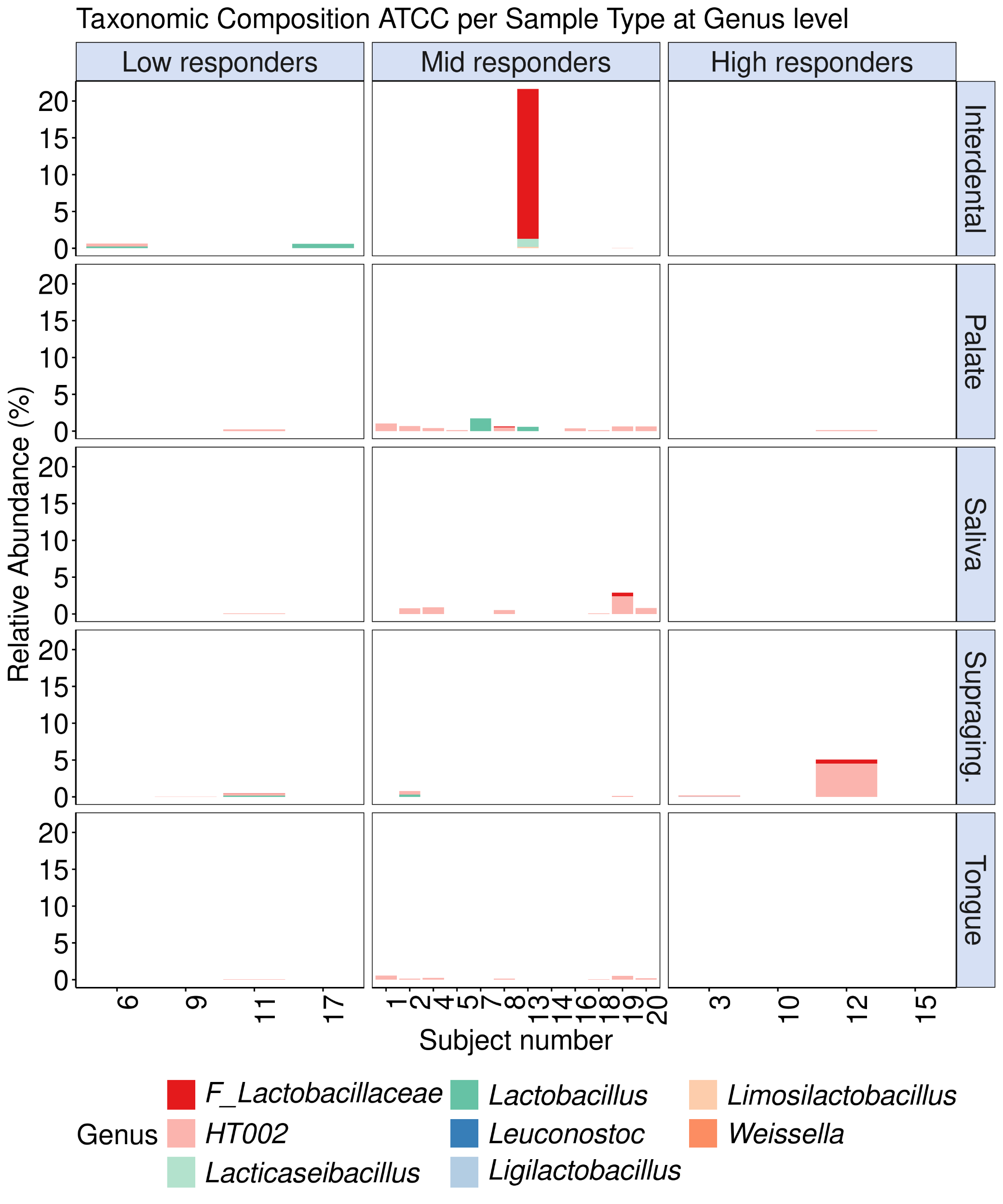
**Supplementary figure 8:** Abundances in all sampling sites of genera of the *Lactobaciliaceae* family per individual in the retainer types for LATCC at day 28.


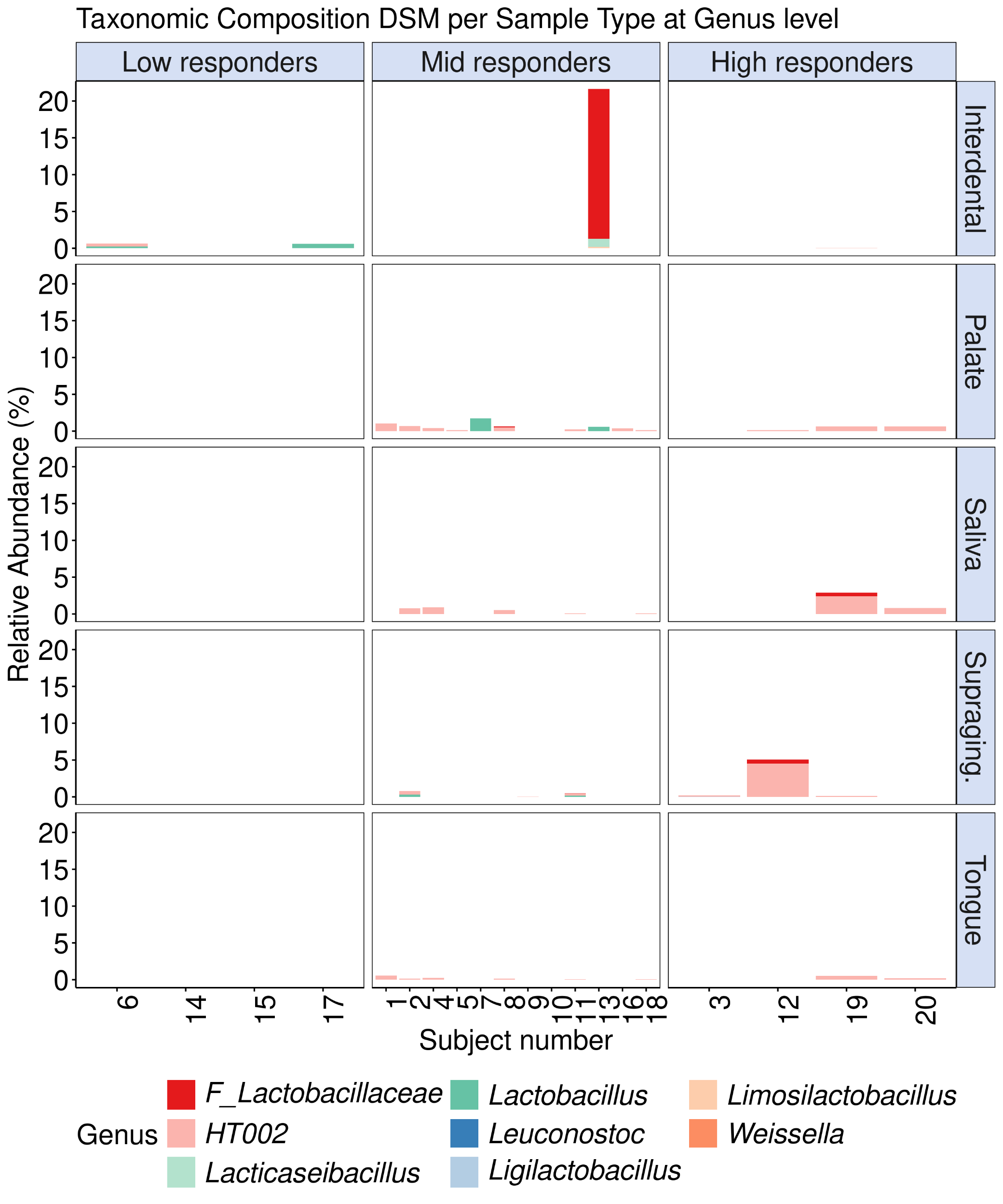
**Supplementary figure 9:** Abundances in all sampling sites of genera of the *Lactobaciliaceae* family per individual in the retainer types for LDSM at day 28.


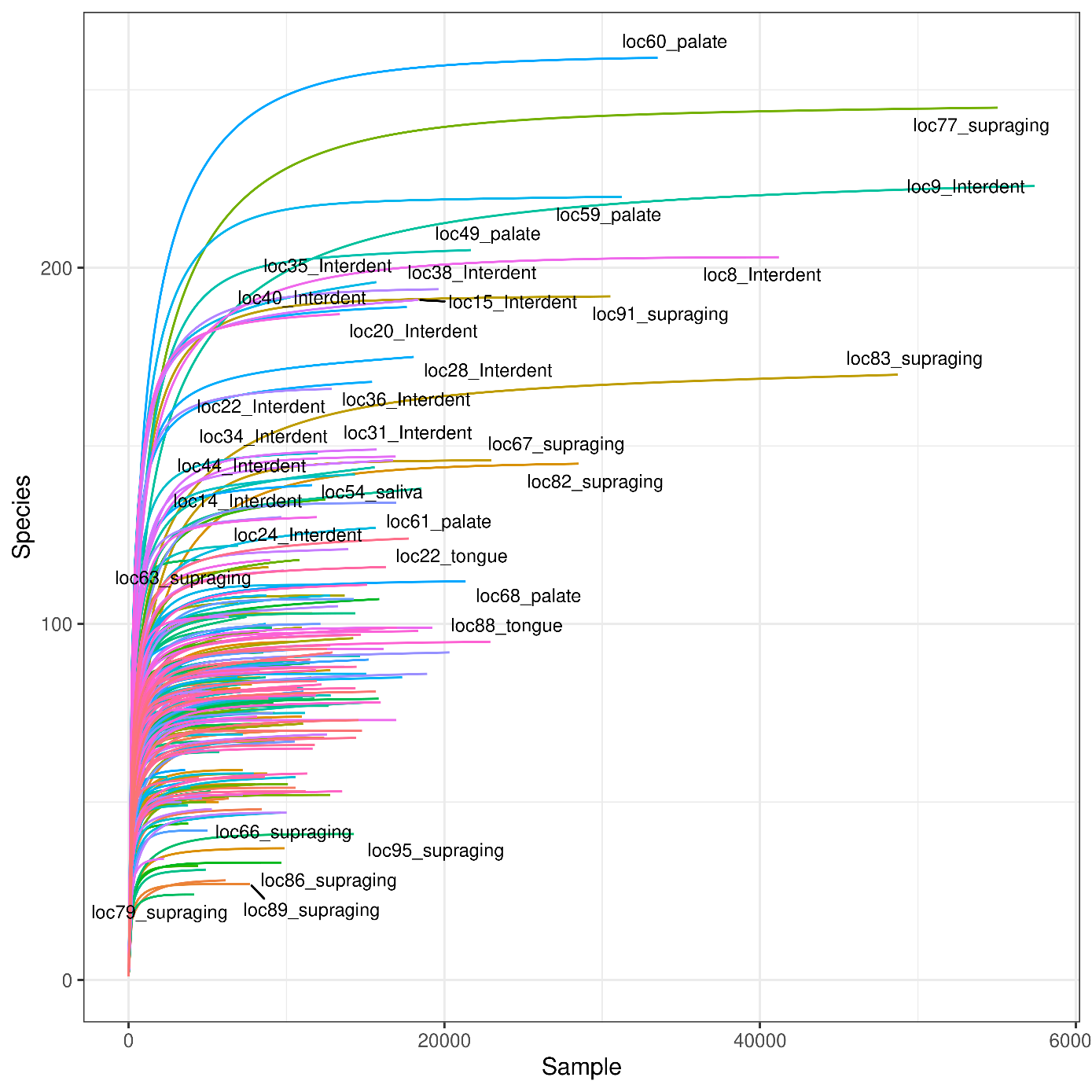


**Supplementary figure 10:** Rarefaction curves of all samples

**Supplementary table 1**: Effect sizes, confidence intervals, and p values of figure 1.

**Supplementary table 2**: Effect sizes, confidence intervals, and p values of figure 2A, for total bacteria.

**Supplementary table 3**: Effect sizes, confidence intervals, and p values of figure 2A, for LATCC.

**Supplementary table 4**: Effect sizes, confidence intervals, and p values of figure 2A, for LDSM.

**Supplementary table 5**: Effect sizes, confidence intervals, and p values of figure 2B&C.

**Supplementary table 6**: Effect sizes, confidence intervals, and p values of figure 6A&B.

**Supplementary table 7**: Effect sizes, confidence intervals, and p values of figure 6C.
